# Double bond configuration of palmitoleate is critical for atheroprotection

**DOI:** 10.1016/j.molmet.2019.08.004

**Published:** 2019-08-07

**Authors:** Ismail Cimen, Zehra Yildirim, Asli Ekin Dogan, Asli Dilber Yildirim, Ozlem Tufanli, Umut Inci Onat, UyenThao Nguyen, Steven M. Watkins, Christian Weber, Ebru Erbay

**Affiliations:** 1 Institute for Cardiovascular Prevention, LMU Munich, German Cardiovascular Research Centre (DZHK), Partner Site Munich Heart Alliance Munich, 80336, Germany; 2 Department of Molecular Biology and Genetics, Bilkent University, Ankara, 06800, Turkey; 3 National Nanotechnology Center, Bilkent University, Ankara, 06800, Turkey; 4 Department of Biomedical Sciences, Cedars-Sinai Medical Center, Los Angeles, CA, 90048, USA; 5 Smidt Heart Institute, Cedars-Sinai Medical Center, Los Angeles, CA, 90048, USA; 6 New York University, Lagone Medical Center, New York, NY 10016, USA; 7 Metabolon, Morrisville, NC, 27560, USA; 8 Verso Biosciences, San Francisco, CA, 94124, USA; 9 Department of Biochemistry, Cardiovascular Research Institute Maastricht (CARIM), Maastricht University, Maastricht, the Netherlands; 10 David Geffen School of Medicine, University of California, Los Angeles, CA, 90095, USA

**Keywords:** Lipid-induced inflammation, Lipokines, Palmitoleate, Ruminant trans-fatty acids, Organelle stress, Inflammasome, Atherosclerosis

## Abstract

**Objective:**

Saturated and trans fat consumption is associated with increased cardiovascular disease (CVD) risk. Current dietary guidelines recommend low fat and significantly reduced trans fat intake. Full fat dairy can worsen dyslipidemia, but recent epidemiological studies show full-fat dairy consumption may reduce diabetes and CVD risk. This dairy paradox prompted a reassessment of the dietary guidelines. The beneficial metabolic effects in dairy have been claimed for a ruminant-derived, trans fatty acid, trans-C16:1n-7 or trans-palmitoleate (trans-PAO). A close relative, cis-PAO, is produced by *de novo* lipogenesis and mediates inter-organ crosstalk, improving insulin-sensitivity and alleviating atherosclerosis in mice. These findings suggest trans-PAO may be a useful substitute for full fat dairy, but a metabolic function for trans-PAO has not been shown to date.

**Methods:**

Using lipidomics, we directly investigated trans-PAO's impact on plasma and tissue lipid profiles in a hypercholesterolemic atherosclerosis mouse model. Furthermore, we investigated trans-PAO's impact on hyperlipidemia-induced inflammation and atherosclerosis progression in these mice.

**Results:**

Oral trans-PAO supplementation led to significant incorporation of trans-PAO into major lipid species in plasma and tissues. Unlike cis-PAO, however, trans-PAO did not prevent organelle stress and inflammation in macrophages or atherosclerosis progression in mice.

**Conclusions:**

A significant, inverse correlation between circulating trans-PAO levels and diabetes incidence and cardiovascular mortality has been reported. Our findings show that trans-PAO can incorporate efficiently into the same pools that its cis counterpart is known to incorporate into. However, we found trans-PAO's anti-inflammatory and anti-atherosclerotic effects are muted due to its different structure from cis-PAO.

## Highlights

• The dairy fat paradox: full-fat dairy consumption associates with lower CVD risk.
• Cis-PAO prevents inflammasome activation and atherosclerosis.
• Trans-PAO, a biomarker for dairy fat consumption, doesn't prevent atherosclerosis.
• The metabolic benefits are muted in trans-PAO due to its specific structure.

## 1 Introduction

Diet is a significant modifier of cardiovascular (CVD) and diabetes risk. The health effects of dietary fats are researched extensively and findings from these studies have been shaping the dietary guidelines. Compelling evidence suggests that a significant reduction in CVD risk can be achieved by replacing saturated fatty acids (SFA) with mono- or poly-unsaturated fatty acids (MUFA or PUFA, respectively) [1]. Moreover, studies consistently associate industrial trans fatty acids (TFA), fatty acid isoforms bearing a trans configuration at the double bond, with a higher CVD risk [2], [3], [4]. Industrial TFA has also been linked to other complex diseases like diabetes, fatty liver disease, stroke, Alzheimer's disease, and cancer [3], [5], [6], [7], [8], [9]. These negative associations have led to the assumption that TFA intake, regardless of its source and type, increases disease risk. However, different TFA subtypes or isomers can be obtained from a variety of dietary sources and they could have divergent metabolic effects [10]. One example is *trans*-palmitoleate (*trans*-16:1n-7 or trans-PAO), a naturally-occurring TFA in full fat dairy, which has been associated with beneficial metabolic effects [11], [12], [13]. Increased circulating ruminant-derived trans-PAO levels mostly reflect dairy fat consumption, which has been associated with lower blood glucose levels, increased insulin sensitivity and overall a lower risk of type II diabetes [13], [14]. More recently, trans-PAO was associated with improved lipid profiles and lower CVD mortality and sudden cardiac death risk [4]. However, the notion that trans-PAO represents a beneficial type of trans-fat remains highly controversial and requires direct experimental evidence [11], [12], [13], [15], [16], [17]. While trans-PAO's role in CVD is debated, its cis isoform has been associated with beneficial metabolic changes in humans and these metabolic benefits have been experimentally demonstrated in mice [18], [19], [20], [21], [22], [23], [24]. Circulating cis-PAO produced by *de novo* lipogenesis (DNL) from the adipose has been shown to mediate inter-organ communication [25]. The beneficial metabolic effects of cis-PAO in high fat-fed mice include increased insulin sensitivity, suppressed hepatic gluconeogenesis, reduced vascular inflammation and atherosclerosis [12], [19], [20], [22], [23], [25]. It remains to be demonstrated that the two isomers of PAO impact immune-metabolic homeostasis and atherosclerosis in the same way.

The primary impact of cis-PAO on atherosclerosis appears to be mediated by its anti-inflammatory actions and especially through preventing inflammasome activation [23]. Driven by hyperlipidemia, atherosclerosis is a chronic inflammatory disease impacting the blood vessel walls [26], [27]. Activation of the Nod-like receptor family, pyrin domain-containing 3 (NLRP3) inflammasome complex in macrophages by cholesterol crystals or saturated fatty acids (SFA), which can be found in these lesions, plays a crucial role in driving a chronic inflammatory response and atherogenesis [28], [29], [30]. The inflammasome complex consists of NACHT, LRR, and PYD domains–containing protein (NALP), apoptosis-associated speck-like protein containing a caspase-1 recruitment domain (ASC), and caspase-1. Their assembly results in proteolytic cleavage of dormant pro-caspase-1 into active caspase-1, which transforms the precursors forms of IL-1β and IL-18 into mature and bioactive cytokines [23], [29], [31]. These cytokines can recruit innate immune cells to the site of infection and modulate adaptive immune cells to drive atherogenesis [31]. Cis-PAO can effectively reduce IL-1β and IL-18 in hyperlipdemic mice [23].

Inflammasome activation can occur in response to organelle stress such as endoplasmic reticulum (ER) and mitochondrial stress. ER stress can be triggered by disruption of ER functions (such as protein folding and calcium homeostasis), but also by excessive lipid flux (such as cholesterol or saturated fatty acids) into cells [32], [33], [34], [35]. ER stress activates the inflammasome through various mechanisms including mobilization of intracellular calcium and the release of reactive oxygen species [23], [36]. Moreover, ER stress propagates mitochondrial oxidative stress and induces mitochondrial ROS (mtROS) production. In most metabolic stress conditions, ER and mitochondrial oxidative stress are intertwined and propagate each other [32], [37]. Just like inflammasome activation, stress in these two organelles plays a causal role in atherosclerosis [23], [29], [30], [36], [38], [39].

In response to ER stress, an elaborate, adaptive signaling is initiated from ER membranes, known as the Unfolded Protein Response (UPR), to re-establish homeostasis in the ER. Three UPR signaling branches are initiated by the ER stress sensors known as inositol-requiring enzyme-1 (IRE1), protein kinase RNA-like endoplasmic reticulum kinase (PERK) and activating transcription factor-6 (ATF6). UPR initiates a gene expression program that up-regulates the expression of chaperones and components of the ER-associated degradation pathway to promote protein folding or to remove misfolded proteins, respectively. Moreover, general protein translation is attenuated to reduce the protein load in the ER lumen [40], [41]. In addition to proteotoxic stress, high fat diets and hyperlipidemia can induce ER stress and activate of UPR pathways *in vivo* in mice and humans, playing a role in the pathogenesis of many complex, inflammatory and metabolic diseases [42], [43], [44]. Whereas in proteotoxic conditions, unfolded proteins activate IRE1 and PERK by recruiting away the glucose-regulated protein 78 (GRP78) from binding these UPR stress sensor's luminal domain, in lipotoxic conditions, the increased lipid levels are instead sensed by their transmembrane domains, inducing IRE1 and PERK oligomerization and activation through auto-phosphorylation [44], [45], [46], [47], [48]. Studies show that inhibition of UPR signaling arms initiated by either IRE1 or PERK prevents inflammasome activation and atherosclerosis in mice [36], [49], [50]. IRE1 and PERK arms have been shown to contribute to inflammasome activation by propagating mitochondrial oxidative stress and mtROS release [36], [49]. Oral supplementation with cis-PAO inhibits both hyperlipidemia-induced UPR signaling and NLRP3 inflammasome activation in macrophages in plaques, resulting in reduced atherosclerosis in mice. Mechanistically, cis-PAO supplementation leads to dynamic integration of this bioactive fatty acid into ER membranes in macrophages and tissues in mice, and prevents high fat-induced adverse remodeling of organelle membranes and subsequent UPR activation [23].

These findings have resulted in a great excitement that dietary supplementation with cis-PAO could prevent CVD. Moreover, recent human epidemiological studies suggest trans-PAO could mediate the beneficial metabolic effects that are associated with high fat dairy consumption. To date, there is no information about trans-PAO's impact on organelles or inflammation [11], [12], [13]. Here, we directly investigated trans-PAO's impact on lipid-induced inflammation and atherosclerosis in a hypercholesterolemic mouse model. Oral supplementation with trans-PAO in mice resulted in its dynamic integration into the major lipid species found in plasma and tissues. Trans-PAO supplementation did not prevent nor augment lipid-induced ER stress, inflammasome activation and atherosclerosis. These findings demonstrate that the cis double bond configuration is crucial for PAO's ability to remodel ER membranes and prevent organelle stress and inflammation, which underlies cis-PAO's atheroprotective action. Therefore, our findings on the two PAO isoforms provide critical guidance for future clinical trials that will continue to address PAO's potential for CVD prevention in humans.

## 2 Materials and methods

### 2.1 General study design

All cell culture experiments were performed as three independent biological replicates. Quantitative reverse transcript polymerase chain reaction (qRT-PCR) analysis was performed from four biological replicates. All the mouse experiments were performed as independent cohorts. The analysis of the *in vivo* experiments (such as atherosclerotic lesion analysis, or immunoflurescent and immunohistochemical staining quantifications) were preformed blind to sample identity. The only elimination criteria for the *in vivo* experiments was based on visible health changes mice and determined by the veterinarian.

### 2.2 Reagents and plasmid

Tissue culture reagents: l-glutamine, DMEM, PBS, HBSS, RPMI, penicillin/streptomycin (P/S), fetal bovine serum (FBS) and RPMI were obtained from Thermo Scientific HyClone. Trypsin-EDTA and HEPES were obtained from GIBCO. Ultrapure LPS, fatty acid-free BSA, palmitate, cis-palmitoleate, NaCl, EDTA, NaF, Triton, sodium orthovanadate (Na_3_VO_4_), phenylmethanesulfonylfluoride (PMSF), and phosphatase inhibitor cocktail-3 were obtained from Sigma Aldrich. Trans palmitoleate was obtained from NuCheck Prep. Primary antibodies used: P-PERK Thr980 (3179), PERK (3192), p-AMPK (2535), total AMPK (2532) from Cell Signaling Technologies; p-eIF2α (144-28G) from Invitrogen; p-IRE1 and IL-1β (ab9722) from Abcam; β-Actin (sc-47778), Caspase-1(sc-514) from Santa Cruz Biotechnology. ECL Prime Western Blot Detection Kit was purchased Amersham Pharmacia and Trisure reagent was purchased from Bioline. The pCMV-Myc-GFP-KDEL plasmid and pcDNA5- IRE1-3F6HGFP-FRT plasmid were obtained from Peter Walter (UCSF) [51].

### 2.3 Cell cultures and treatment

#### Mouse BMDM isolation

BMDM were isolated from the tibiae and femurs of mice and differentiated for five to seven days in RPMI-1640 growth medium enriched with %15 L929 cells (ATCC) conditioned medium. The differentiated BMDM cells were primed with 200 ng ultrapure LPS for 3 h, Then, cells were stimulated with ethanol-BSA (negative control), palmitate-BSA (500 μM, cis-palmitoleate-BSA (500 μM) or trans-palmitoleate-BSA (500 μM) for the indicated amounts of time. Proteins (from cells or conditioned medium) and RNA were isolated from the cells as described earlier [23].

#### Fatty acid-Bovine albumin serum complex

Palmitate (500 mM in ethanol) was prepared as previously described and added to the cell media at indicated dose [49].

### 2.4 Protein analysis

Protein lysates were prepared as described before [23], [36]. All lysates were run on SDS-PAGE gels, transferred to PVDF membranes, before blocking (in TBS with 0.1% Tween-20 (v/v) and 5% (w/v) dry milk or BSA). For cleaved caspase-1 and IL-1β detection: the conditioned medium was collected and mixed with 5X SDS loading dye, followed by heating at 95 °C for 5 min before loading on SDS-PAGE gels. After secondary-HRP conjugated antibody incubation, all blots were developed using ECL prime reagent (GE Healthcare) and visualized by BioRAD MP imager.

### 2.5 RNA isolation and qRT-PCR

Total RNA was extracted with Trisure reagent (Bioline) and converted to cDNA with Revert Aid First Strand cDNA Synthesis Kit (Thermo Scientific). Using specific primers, cDNAs were amplified on Light Cycler 480II (Roche) or Rotor Gene (Qiagen). The formula that was used for calculating expression changes is as follows: (primer efficiency)^−ΔΔCt^ where ΔΔCt means ΔCt (target gene) - ΔCt (reference gene) and Ct means (threshold cycle). Results are representative from three or more independent experiments that were quantified and analyzed by Student's t-test.

The following primers were used

mTNFα-Frw:5′-CATCTTCTCAAAATTCGAGTGACAA-3′;

mTNFα-Rev:5′-TGGGAGTAGACAAGGTACAACCC-3′;

mCCL2-Frw: 5′- CTTCTGGGCCTGCTGTTCA-3′

mCCL2-Rev: 5′-CCAGCCTACTCATTGGGATCA-3′

mGAPDH-Frw:5′-GTGAAGGTCGGTGTGAACG-3′;

mGAPDH-Rev: 5′- GGTCGTTGATGGCAACAATCTC -3′.

### 2.7 mtROS staining and quantification

For staining of mtROS production, MitoSOX™ Red mitochondrial superoxide indicator (Life Technologies) was used as previously described [36]. Mitochondrial stainings were done with Mitotracker GreenFM (Life Technologies) according to the manufacturer's instructions. The images were analyzed with ImageJ program. The images were taken with Leica DMI 4000B equipped with Andor DSD2 spinning disk confocal microscope (Cagdas Son Lab, METU).

### 2.8 IRE1 oligomerization

IRE1-3F6HGFP expressing HEK-293 stable cell lines were used as published [23]. 1 × 10^6^ cells HEK-293 stable cells on cover slips were treated with BSA (control), palmitate-BSA (500 μM), cis-palmitoleate-BSA (500 μM) or trans-palmitoleate-BSA (500 μM) alone or in combination for 6 h. Images of the cells were acquired on Leica DMI 4000B equipped with Andor DSD2 spinning disk confocal microscope (Cagdas Son Lab, Middle East Technical University, Ankara, Turkey). Oligomerization analysis was performed according to previously published protocols by counting IRE1 foci in >100 cells from multiple replicates for each treatment [23].

### 2.9 Cytokine measurements

To quantify IL-1β amount in plasma, samples were diluted 1:1 using assay diluent A and ninety-six-well colorimetric “sandwich” ELISA plates (Abcam, mouse IL-1β ELISA kit) was used: Recombinant mouse IL-1β standard (100 μl of 2000–2.74 pg/ml) and 100 μl of diluted plasma samples were run in duplicate according to manufacturer's instructions. The absorbance was detected at 450 nm using plate readers. Results were calculated by Prism 8.1.2 (GraphPad Software Inc.).

### 2.10 Mouse models, dietary treatments, and experimental procedures

Animal care and experimental procedures were performed according to the local animal care and ethical review committee guidelines accepted at İhsan Dogramaci Bilkent University. For our studies, Apoe^−/−^ mice (Charles River) were used. Male mice were fed with Western diet from the age of week 8 for 12 weeks. Then, the mice were treated by oral gavage with vehicle (1% BSA in PBS) or 1400 mg/kg/day cis or trans-PAO dissolved in vehicle for 4 weeks while continuing on Western diet. The mice weights were measured weekly and blood glucose measurements were done at the beginning and at the end of injections before sacrifice. The mouse sacrifice and tissue collection was described in detail in our previous publications [23], [36].

### 2.11 En face aorta lesion analysis

Aortas were pinned on a black wax surface, and atherosclerotic lesions were analyzed in all aorta after Sudan IV staining as described earlier [23]. Lesion area was quantified using ImageJ and expressed as the percentage of SUDAN IV stained plaque area over the aortic area.

### 2.12 Plasma lipids and lipoprotein analysis

Mouse blood was obtained via heart puncture during sacrifice, centrifuged and plasma collected. Then, the plasma was analyzed by FPLC in the Mouse Metabolic Phenotyping Center at the University of Cincinnati. For the resolution of major lipoprotein classes from plasma, the columns were equilibrated in 50 mM PBS. Using a microtiter plate enzyme-based assay, the major lipoprotein classes were measured in cholesterol or triglyceride assays from collected fractions.

### 2.13 Lipidomic analysis

Lipidomic analysis of plasma and tissues including muscle, liver, and adipose tissue (control; n = 5, trans-PAO; n = 5, and cis-PAO; n = 6) were done by Metabolon. The analysis was performed as previously described [18], [23]. Individual lipid species were quantified by taking the peak area ratios of target compounds and their assigned internal standards, then multiplying by the concentration of internal standard added to the sample. Lipid class concentrations were calculated from the sum of all molecular species within a class, and fatty acid compositions were determined by calculating the proportion of each class comprised of individual fatty acids.

Quantitative analysis were for the following: (A) lipid classes: FFA: free fatty acid, CE: cholesteryl ester, DAG: diacylglycerol, TAG: triacylglycerol, CL: cardiolipin, LYPC: lysophosphatidylcholine, PC: phosphatidylcholine, PE: phosphatidylethanolamine, PS: phosphatidylserine. (B) fatty acids: 14:0, 15:0, 16:0, 18:0, 20:0, 22:0, 24:0, 14:1n5, 16:1n7, 18:1n7, 18:1n9, 20:1n9, 20:3n9, 22:1n9, 24:1n9, 18:2n6, 18:3n6, 20:2n6, 20:3n6, 20:4n6, 22:2n6, 22:4n6, 22:5n6, 18:3n3, 18:4n3, 20:3n3, 20:4n3, 20:5n3, 22:5n3, 22:6n3, 24:6n3, plasmalogen derivatives of 16:0, 18:0, 18:1n7, 18:1n9, t16:1n7, t18:1n9, and t18:2n6.

### 2.14 Immunohistochemistry

7-μm thick cryosections were obtained using a cryostat (Leica CM 1850) from the aortic roots. Immunofluorescent stainings were performed using the following antibodies: anti–MOMA-2 (monocyte/macrophage marker) (abcam ab33451), anti–CD3-Alexa488 (Biolegend 100–210), anti–α-SMA (abcam ab5694), anti-P-eIF2α (Invitrogen 144-28G), anti-ATF3 (sc-188; Santa Cruz Biotechnology), IL-1β antibody (abcam ab9722) and DAPI (abcam ab104140). Cryosections were stained with Masson's Trichrome (Bio-Optica), OilRedO (Sigma), FAM/FLICA caspase1 detection kit (Immunochemistry Tech FAM-YVAD-FMK(655) # 97), TUNEL (In situ Cell Death detection Kit, Fluorescenin; Roche 11684795910) and hematoxylin and eosin (H&E). Representative images were taken with a Zeiss fluorescent microscope.

Cryosections were stained with H&E stain for morphometric lesion analysis. The total lesion area and necrotic area were quantified using ImageJ software as previously described from 4 sequential sections (60 μM apart, beginning at the base of the aortic root) [23], [52], [53], [54]. Foam cell area was calculated from OilRedO stained 4 sequential sections and collagen content from Masson's Trichrome stained sections using ImageJ as previously described [23].

The fluorescent immunostainings were carried out on cryosections that were fixed in cold acetone for 10 min, blocked in %4–6 BSA/PBS with %10–20 species specific serum (compatible with the primary antibody) as previously described [49]. All stained sections were mounted with Fluorosave mounting reagent containing DAPI. Florescent signal calculations: (a) ATF3, P-eIF2α, and FAM/FLICA stainings: the sections were double stained with MOMA-2 to mark the macrophage-enriched area. The Mean Fluorescent Intensity (MFI) corresponding to primary antibody signal was calculated from this MOMA-2 positive area. The background fluorescence of the non-stained area inside the lesion was subtracted from the total MFI corresponding to each signal. Data were quantified as total MFI signal compared with baseline [22], [55], [56]. (b) CD3: The total T cell number (cells/mm2) to the lesion area was quantified from CD3 as previously described [23] (c) other fluorescent stainings was calculated as percentage of MOMA-2, IL-1β, and α-SMA positive-stained area over total lesion area [23], [36].

### 2.15 Statistical analysis

Results are reported as mean ± SEM. Statistical significance of the results was determined using the Student's t test, one-way ANOVA (for more than two groups) or the Mann–Whitney test (for *in vivo* analysis). P < 0.05 was considered as significant.

## 3 Results

### 3.1 The impact of trans-PAO on lipid-induced ER stress and inflammation in macrophages

**Figure 1:**
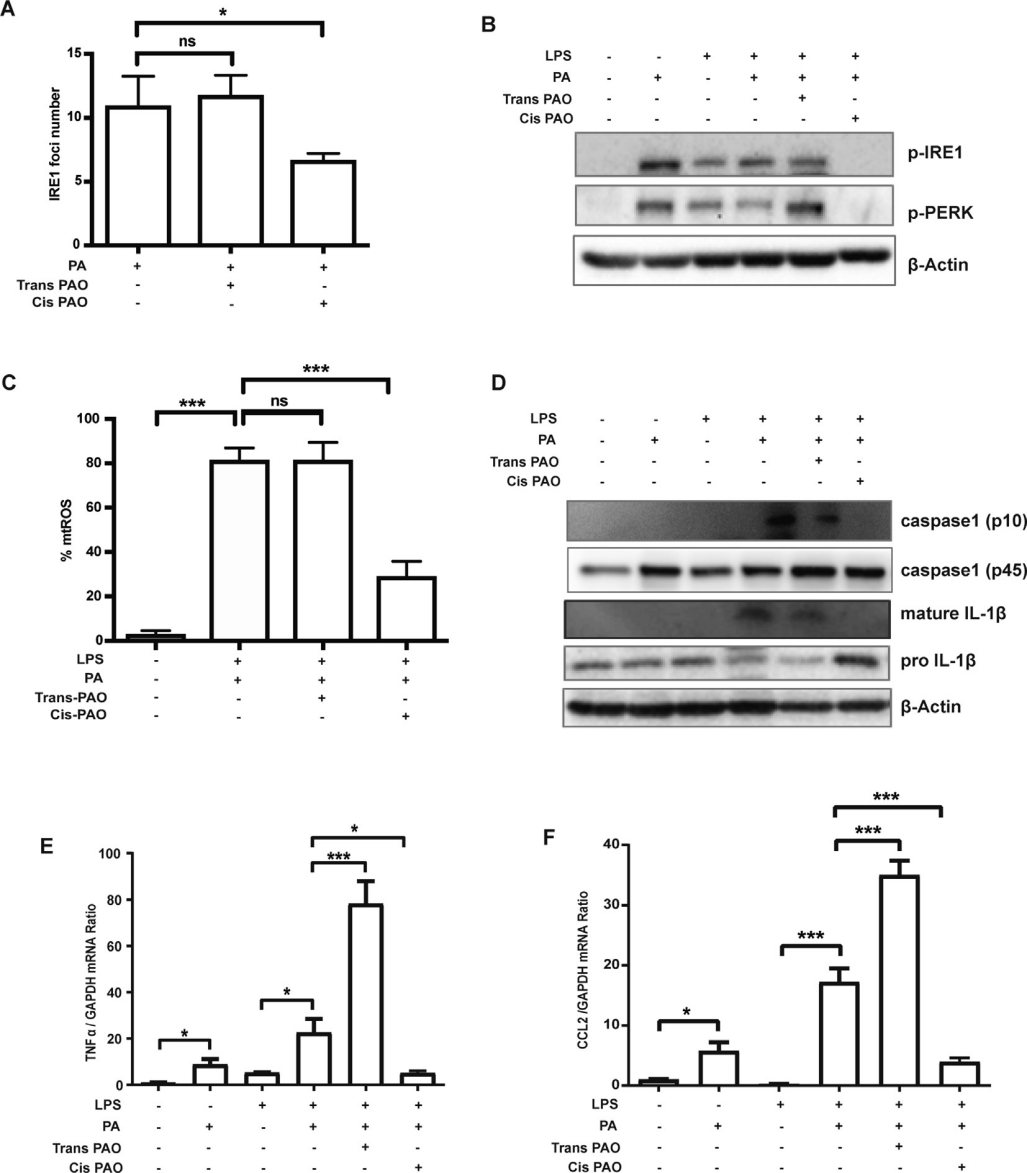
**Trans-PAO treatment does not prevent lipid-induced organelle stress or inflammation in mouse macrophages. (A)** Cells co-transfected with a green fluorescent protein (GFP)-tagged IRE1 plasmid and Cherry-KDEL amino acid sequence expressing construct were treated with 500 μMPA or co-treated with 500 μMPA and 500 μM trans-PAO/cis-PAO for six hours. IRE1 oligomer/foci formation (green) on ER membranes (red) was analyzed by confocal microscopy. The graph displays quantification of IRE1 oligomerization (distinct IRE1 foci per cell). Data represents mean ± SEM; **P < 0.01, n ≥ 100 from four experiments. **(B)** LPS-primed and PA-stressed BMDM were co-treated with trans-PAO or cis-PAO and protein lysates were analyzed by western blotting using specific antibodies against: p-PERK, p-IRE1 and β-actin. **(C)** LPS-primed and PA-stressed BMDM were treated with cis- or trans-PAO and mtROS production was measured with MitoSOX™ (red mitochondrial superoxide indicator kit) (n ≥ 3). (**D**) LPS-primed and PA-stimulated BMDM were co-treated with cis- or trans-PAO and the conditioned medium was analyzed by western blotting using specific antibodies against IL-1β, caspase-1 and β-actin (n ≥ 3). (**E-F**) qRT-PCR analysis (**E**) TNFα and (**F**) CCL2 mRNA in LPS-primed, PA-stressed BMDMs co-treated with cis- or trans-PAO (n ≥ 3). Data are shown as means ± SEM. *P < 0.05,**P < 0.01,***P < 0.001. One way ANOVA was used for statistical analysis.

Cholesterol, phospholipids, and the fatty acid content in these lipids determine the biophysical properties of membrane systems, including the ER. The lipid composition of the ER membranes is sensed through the transmembrane domains of the ER stress sensors and determines their activation status [23], [48]. Previously, we showed that SFA can alter macrophage ER membrane lipid composition and initiate UPR signaling such as by inducing IRE1 oligomerization on the ER membranes [23]. SFA-induced UPR signaling can be counteracted by cis-PAO, which enters and remodels ER membranes [23]. Here, we assessed the impact of trans-PAO on IRE1 oligomerization on the ER membranes and compared to cis-PAO. As expected, cis-PAO treatment significantly reduced IRE1 oligomer formation (as assessed by visualizing green fluorescent protein (GFP)–tagged IRE1 foci formation on ER membranes). However, trans-PAO treatment did not alter PA-induced IRE1 oligomerization on ER membranes (P < 0.05, Figure 1A). While cis-PAO prevented SFA-induced IRE1 activation (as assessed by IRE1 auto-phosphorylation) as expected, trans-PAO treatment did not inhibit lipid-induced IRE1 activation in mouse and human macrophages (Figure 1B, S. Fig. 1A). Similarly, cis-PAO, but not trans-PAO, prevented SFA-induced PERK activation (as assessed by PERK auto-phosphorylation) in mouse macrophages (Figure 1B), demonstrating trans-PAO does not have an impact on lipid-induced ER stress.

Next, we assessed the two PAO isomers' impact on SFA-induced mitochondrial oxidative stress in macrophages side by side. We observed cis-PAO treatment significantly suppressed lipid-induced mtROS generation, but trans-PAO treatment did not (P < 0.001, Figure 1C, S. Fig. 1B). Taken together, our results show trans-PAO can't prevent SFA-induced organelle stress. These findings thus suggest that trans-PAO and cis-PAO may differ in their biological activities such as inflammation and in their role in atheroprotection.

SFA specifically leads to the induction of NLRP3 inflammasome and IL-1β and IL-18 secretion in UPR-dependent manner in both mouse and human macrophages, and this can be blocked by cis-PAO treatment [23], [30], [36], [57], [58]. Therefore, we next investigated whether trans-PAO has an impact on SFA-induced inflammasome activation. We observed that trans-PAO treatment does not abolish PA-induced caspase-1 and IL-1β secretion, which was significantly blocked by cis-PAO in the same experimental set up (Figure 1D). Moreover, trans-PAO co-treatment did not prevent PA from inducing inflammatory cytokines such as tumor necrosis factor-alpha (TNFα) and monocyte chemotactic protein-1 (MCP1)/chemokine (C–C) motif ligand-2 (CCL2) in mouse macrophages, but appeared to augment it (P < 0.001; Figure 1E, F). A previous study suggested that cis-PAO increases oxidative respiration and reduces inflammation in macrophages through activation of AMPK. However, both trans-PAO and cis-PAO treatment increased AMPK phosphorylation in macrophages suggesting this is not the mechanism by which these two PAO isoforms differentially impact lipid-induced inflammation in macrophages (S. Fig. 1C) [59]. These findings demonstrate that unlike cis-PAO, trans-PAO treatment of macrophages does not appear prevent SFA-induced inflammation and it may not impact atherosclerosis in a beneficial way.

### 3.2 *The impact of trans-PAO on lipid composition* in vivo

**Figure 2:**
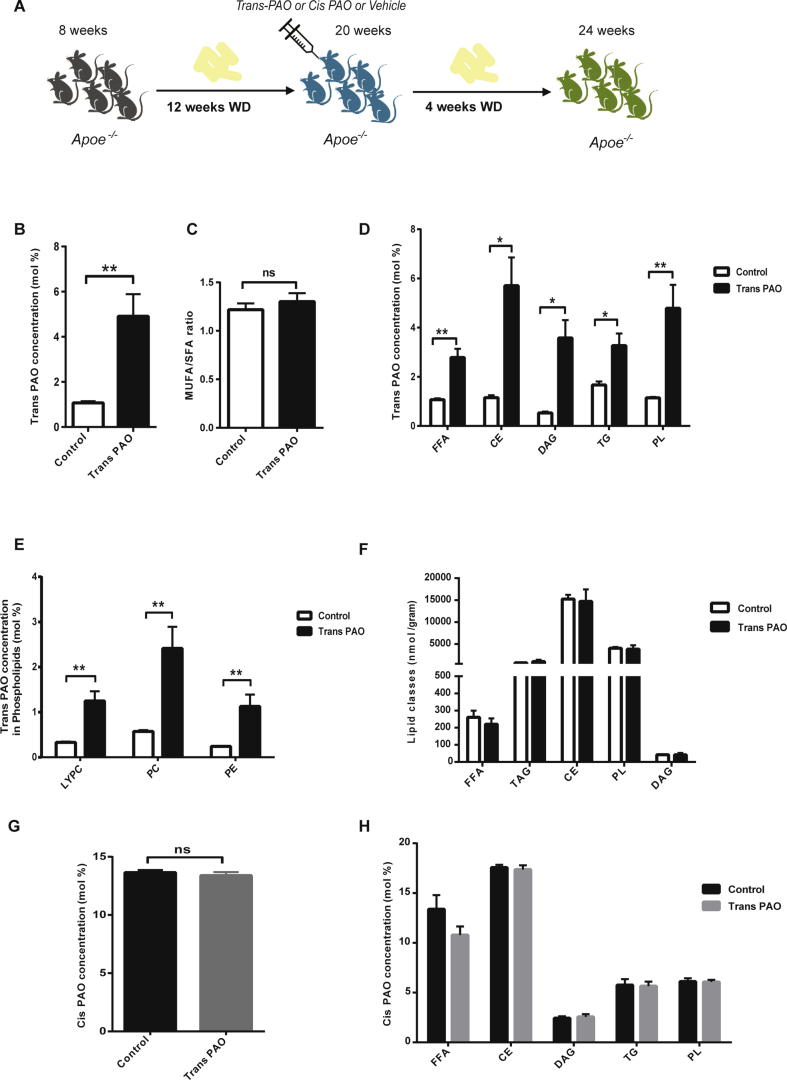
**The impact of trans-PAO treatment on plasma lipid composition in the Apoe**^−/−^**mice. (A)** Experimental design for the analysis of atherosclerosis in Apoe^−/−^ mice on a Western diet (WD). Quantitative lipidomic analysis was performed with plasma isolated from trans-PAO- and vehicle-treated Apoe^−/−^ mice on WD: **(B**) The mean concentration of trans-16:1n-7 (mole %; the ratio of moles of fatty acids to total moles of fatty acids) in plasma. **(C)** MUFA/SFA ratio. **(D)** The mean concentration of trans-C16:1n-7 (mole %) in various lipid classes. **(E)** The mean concentration of trans-C16:1n-7 (mole %) in the various phospholipid classes. (**F**) The mean concentration (mole %) of various lipid classes. (**G**) The mean concentration of cis-C16:1n7 (mole %). (**H**) The mean concentration of cis-C16:1n7 (mole %) in various lipid metabolites. Data represents means ± SEM; *P < 0.05, **P < 0.01, ns = not significant, (n = 5 per group). Unpaired two-tailed Student's t test was used for statistical analysis. (FFA: free fatty acid, CE: cholesteryl ester, DAG: diacylglycerol, TAG: triacylglycerol, CL: cardiolipin, LYPC: lysophosphatidylcholine, PC: phosphatidylcholine, PE: phosphatidylethanolamine, PS: phosphatidylserine).

In contrast to the epidemiological data linking trans-PAO with beneficial outcomes in CVD, our findings in macrophages suggest that trans-PAO does not prevent organelle stress and inflammation in macrophages stimulated with PA *in vitro* and therefore trans-PAO may be devoid of the atheroprotective qualities that are attribute to cis-PAO. We next conducted an *in vivo* study to observe the impact of trans-PAO supplementation on hyperlipidemia-induced inflammation and atherogenesis. For this purpose, 8 week-old male apolipoprotein E-deficient (Apoe^−/−^) mice were fed with Western diet (WD) for 16 weeks to induce hyperlipidemia and atherosclerosis development. Starting at 12 weeks of this diet regimen, we administered 1400 mg/kg/day cis or trans-PAO (based on the atheroprotective cis-PAO dosage determined by our earlier experiments [23]) or vehicle (control group) by oral gavage (Figure 2A; experimental design). Although incorporation efficiency into various lipid classes in tissues varies for different TFA species, dietary TFA can incorporate into serum lipoproteins and adipose tissue in the form of triglyceride, phospholipids, and cholesterol esters [60]. The effect of dietary trans-PAO on plasma and tissue lipid composition has not been studied before. We investigated the efficiency of trans-PAO incorporation into various lipid classes such as free fatty acids (FFA), cholesterol esters (CE), diacylglycerol (DAG), triacylglycerol (TG) and phospholipid (PL) in plasma and multiple tissues by way of quantitative lipidomics analysis. We observed 4 weeks of trans-PAO treatment increased trans-PAO levels in the plasma of Apoe^−/−^ mice (P < 0.01; Figure 2B). Trans-PAO treatment did not cause a change in the plasma MUFA/SFA ratio (Figure 2C). While trans-PAO concentration in FFA, CE, DAG, TG and PL was higher compared to control (P < 0.05; Figure 2D, E), the concentration of these lipid classes was unaltered in the plasma (Figure 2F). Trans-PAO concentration was also increased in many of the PL species without expanding the PL compartment in plasma (P < 0.05; Figure 2D–F). Additionally, trans-PAO treatment did not impact cis-PAO total concentration (Figure 2G) or cis-PAO amount within any major lipid class in plasma (Figure 2H). These findings indicate that oral trans-PAO supplementation effectively increases plasma trans-PAO concentration and trans-PAO incorporation into various lipid compartments in plasma.

**Figure 3:**
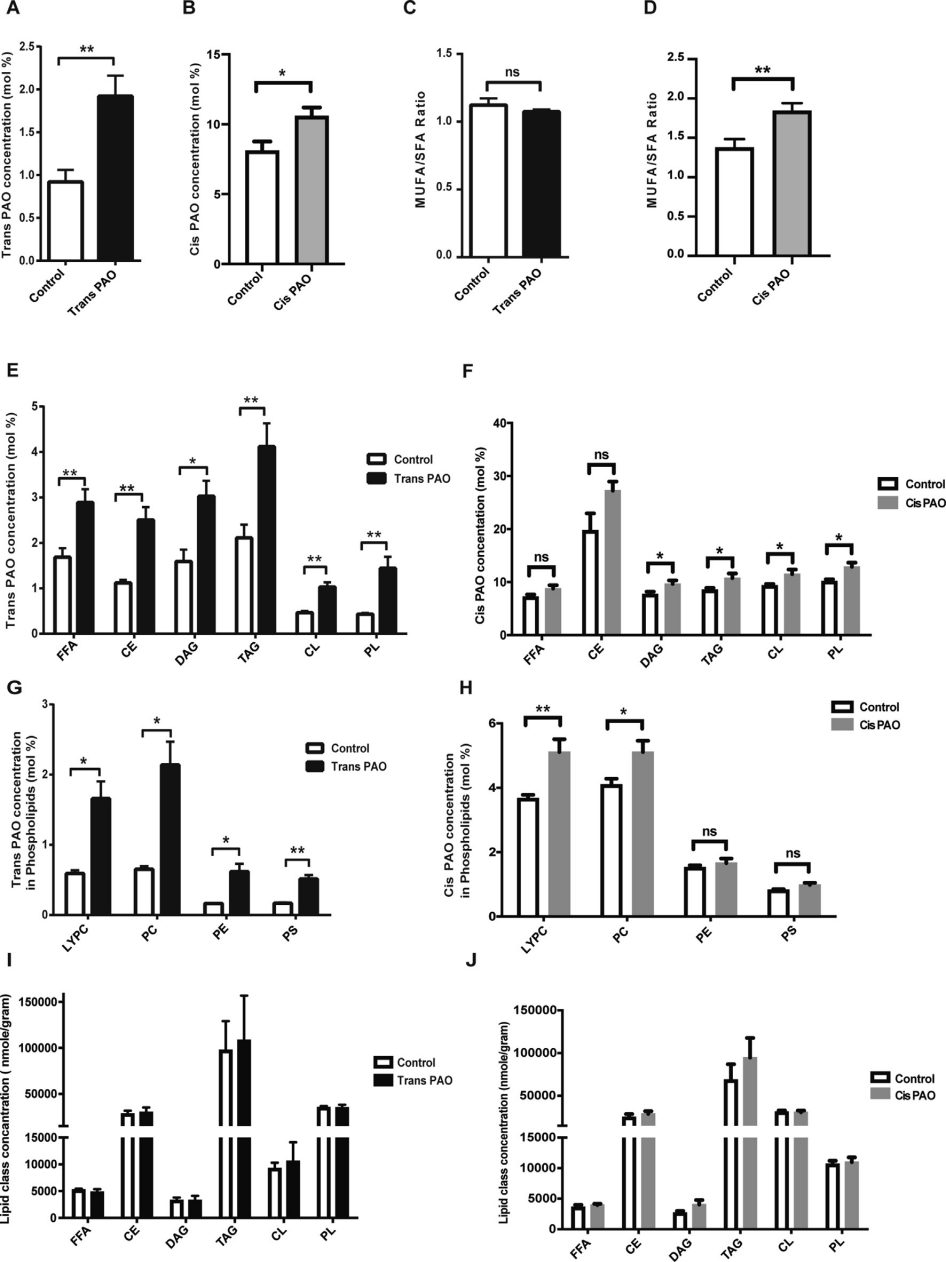
**The effect of trans- or cis-PAO treatments on liver lipid composition in Apoe**^**−/−**^**mice.** Quantitative lipidomic analysis was performed with livers from trans-PAO- or cis-PAO-treated and control Apoe^−/−^ mice on WD: **(A,B)** The mean concentration of trans- or cis-C16:1n-7 (mole %), **(C,D)** MUFA/SFA ratio after trans- or cis-PAO treatment. **(E, F)** The mean concentration (mole %) of trans- or cis-C16:1n-7 in various lipid classes are shown. (**G,H**) The mean concentration (mole %) of trans- or cis-C16:1n-7 in the various phospholipid classes. (**I,J**) The mean concentration (mole/gram) of various lipid classes in trans- or cis-PAO treated and control mice. Data represents mean ± SEM; *P < 0.05, **P < 0.01, ns = not significant, (n = 5 for trans-PAO and n = 6 for cis-PAO). Unpaired two-tailed Student's t test was used for statistical analysis.

Next, we assessed the lipid profiles from liver, skeletal muscle tissue and adipose tissue of trans-PAO, cis-PAO and vehicle-treated Apoe^-/-^ mice. The concentration of trans-PAO or cis-PAO significantly increased in the liver and muscle from the trans-PAO-treated mice or cis-PAO-treated when compared to control (P < 0.05; Figure 3A,B, S. Fig. 2A, B). Similar to plasma, trans-PAO treatment did not alter the MUFA/SFA ratio in liver or muscle (Figure 3C, S. Fig. 2C), whereas cis-PAO treatment significantly increased MUFA/SFA ratio in liver or muscle (Figure 3D, S. Fig. 2D). Trans-PAO amount in the major lipid class (FFA, EC, DAG, TAG, CL, PL) or cis-PAO amount in the major lipid class (DAG, TG, CL, PL) was increased in liver and muscle but without causing an expansion of these lipid classes analyzed in the samples (P < 0.05; Figure 3E–J and S. Fig. 2E–J). The concentration of trans-PAO significantly increased in the adipose tissue, while concentration of cis-PAO did not (P < 0.05; S. Fig. 3A, B). Trans-PAO or cis-PAO treatment did not alter MUFA/SFA ratio in adipose tissue (S. Fig. 3C, D). Trans-PAO amount in the major lipid class (FFA, DAG, TAG, PL) significantly increased in adipose tissue (S. Fig. 3E) but the amount of cis-PAO in these lipid classes was similar (S. Fig. 3F). Furthermore, trans-PAO or cis-PAO treatment did not cause the expansion of these lipid classes in adipose tissue (S. Fig. 3G, H). In conclusion, chronic trans-PAO treatment significantly increased the incorporation of trans-PAO into the major lipid classes (FFA, EC, DAG, TAG, CL, PL) in the tissues but these lipids′ levels or the overall fatty acid desaturation index (MUFA: SFA ratio) remained unchanged. Cis-PAO treatment significantly increased the incorporation of cis-PAO into major lipid classes in the liver (DAG, TAG, CL, PL) and in the muscle (CE, CL, PL), but not in adipose tissue cis-PAO treatment significantly changed the fatty acid desaturation index in liver and muscle but not adipose tissue.

### 3.3 *The impact of trans-PAO on macrophage ER stress and inflammasome activation* in vivo

**Figure 4:**
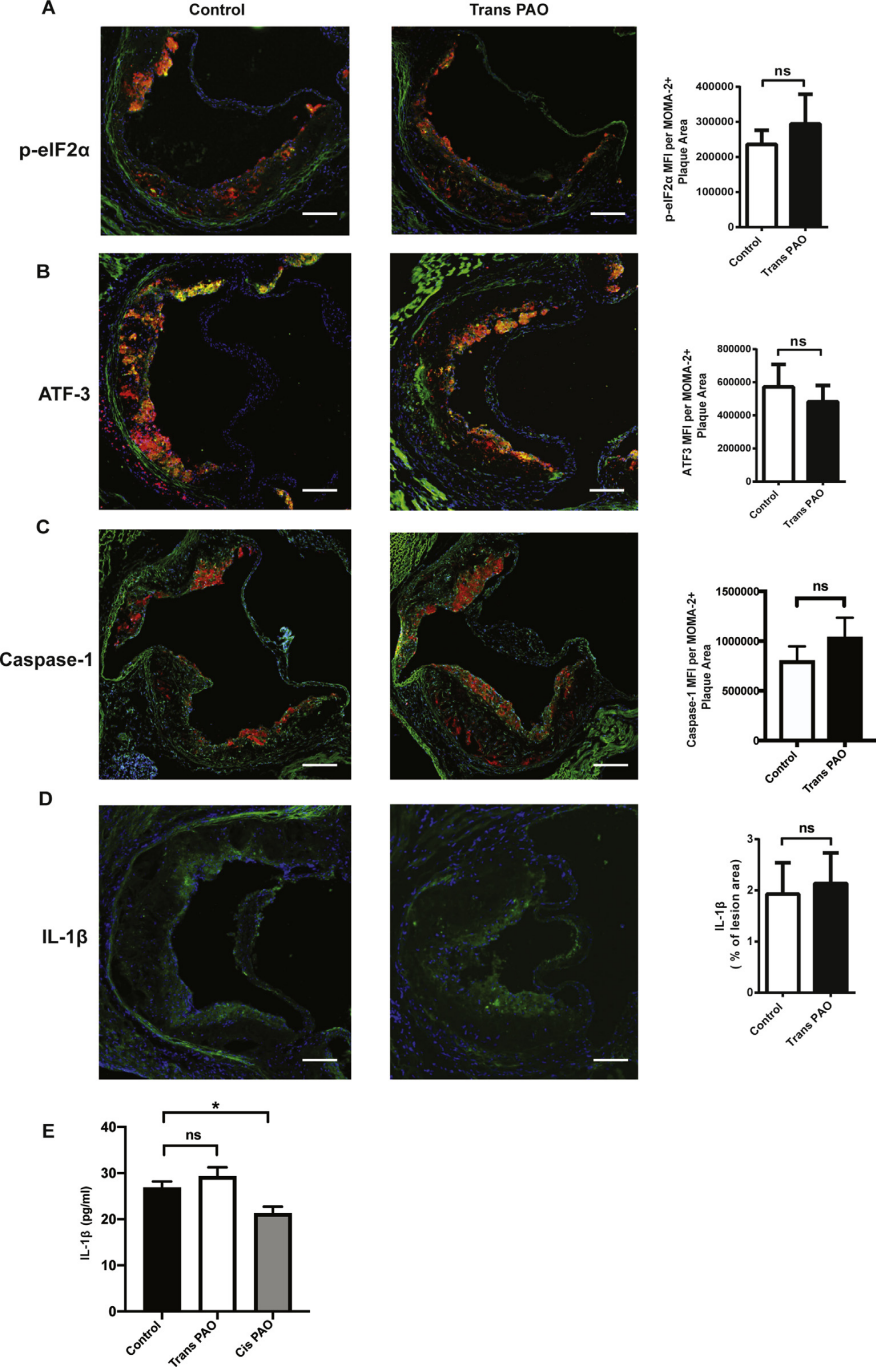
**Trans-PAO does not prevent ER stress or inflammasome activation *in vivo***. Aortic root sections from trans-PAO or vehicle treated mice were analyzed by immunofluorescent (IF) staining using specific antibodies for: (**A)** p-eIF2α (green), **(B)** ATF3 (green), **(C)** FLICA (green), and (**D**) IL-1β (green). For each staining, a representative image is shown in left and center, and the quantification of the data appears on the right. Relative IF intensities (green) for P-eIF2α, ATF3 and FLICA were quantified from the macrophage positive area (red) (n = 5 per group). **(E)** Plasma IL-1β (levels were measured from the Apoe^−/−^ mice treated with trans- or cis-PAO or vehicle for 4 weeks (n = 6 per group). All data represent mean ± SEM; *P < 0.05, **P < 0.01. Control versus trans-PAO (A,B,C,D) or Cis-PAO (E); Mann Whitney U test was used for statistical analysis. Scale bars: 150 μm.

Macrophages form the vast majority of inflammatory cells in atherosclerotic plaques, and they play an important role in the development and progression of atherosclerosis through modulation of cholesterol homeostasis, the immune-inflammatory response, and plaque cellularity [61], [62], [63]. Increased ER stress in lipid-laden macrophages has been connected to plaque progression, vulnerability, and rupture and to CAD in humans, whereas suppression of ER stress alleviates atherosclerosis in mouse models [64]. In addition, decreasing ER stress suppresses inflammasome activation in lesion macrophages [23], [36]. Even though cis-PAO suppressed lipid-induced ER stress and subsequent NLRP3 inflammasome activation [23], trans-PAO could not block SFA-induced IL-1β secretion in macrophages (Figure 1D). Hence, we sought to understand the impact of trans-PAO on ER stress and subsequent inflammasome activation in atherosclerotic plaque macrophages *in vivo*. For this purpose, we stained the macrophage-enriched areas of aortic root and performed immunofluorescence signal intensity analysis for two surrogate markers of UPR, namely phosphorylation of eukaryotic initiation factor 2α (eIF2α) and downstream of eif2α signaling, the induction of the expression of cyclic adenosine monophosphate–dependent transcription factor 3 (ATF3). The data confirmed that trans-PAO treatment does not suppress P-eIF2α or ATF3 expression in macrophage-filled (MOMA2-positive) plaque area when compared to the control mice (Figure 4A,B). To investigate the trans-PAO's impact on inflammasome activation *in vivo*, we performed immunofluorescence staining for IL-1β and active caspase-1 in plaque macrophages. We did not find any differences in immunofluorescence staining for IL-1β and active caspase-1 in the macrophage-enriched (MOMA2 positive) plaque regions (Figure 4C,D). ELISA analysis of the serum from mice treated with trans-PAO showed no significant differences in systemic IL-1β levels between trans-PAO and control treatment mice, whereas cis-PAO significantly reduces systemic IL-1β levels (Figure 4E). These findings show that trans-PAO unlike cis-PAO, does not prevent hyperlipidemia-induced macrophage ER stress and inflammasome activity in plaque areas *in vivo*.

### 3.4 The impact of trans-PAO on atherosclerosis

**Figure 5:**
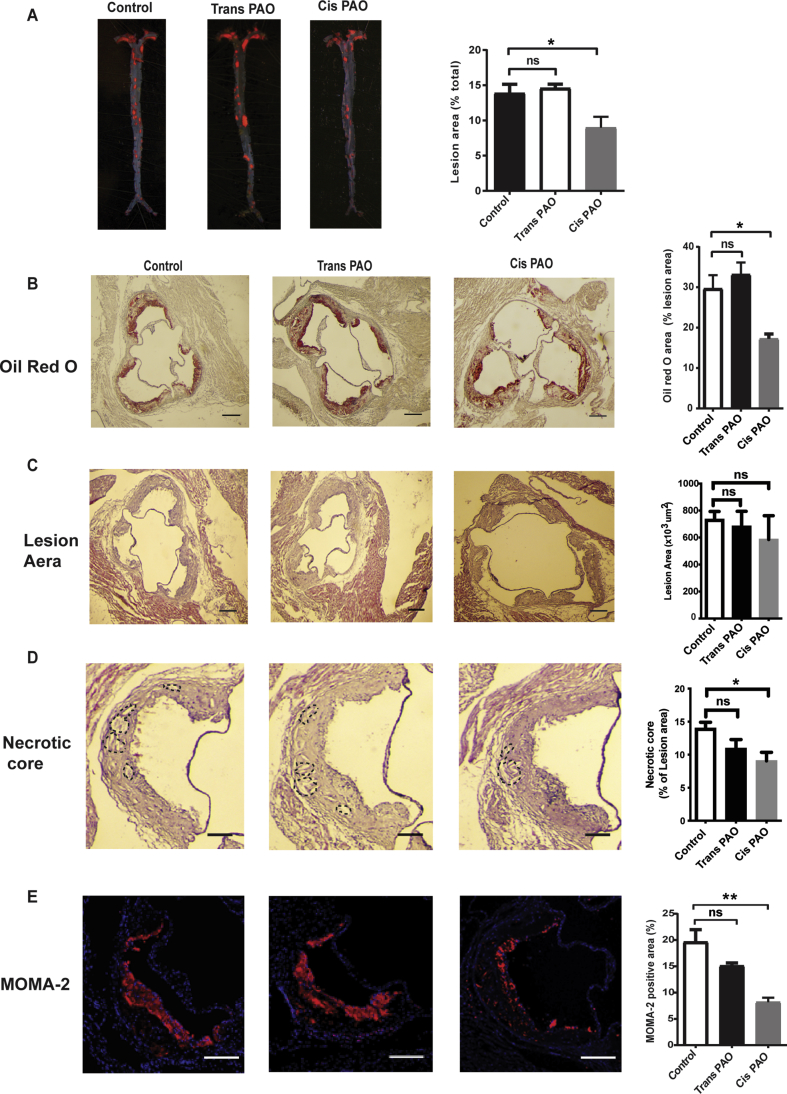
**Trans-PAO does not prevent atherosclerosis in Apoe**^**−/−**^**mice.** (**A**) Lesion area was calculated from en face aorta preparations stained with Sudan IV (Control; n = 12; Trans-PAO; n = 9; Cis-PAO; n = 6). (**B**) Foam cell area was calculated from Oil RedO stained aortic root sections (control; n = 9; Trans-PAO; n = 9, Cis-PAO; n = 5 scale bar: 300 μm). (**C**) Total plaque area and (**D**) necrotic area were calculated from hematoxylin and eosin (H&E)-stained aortic root lesions (control; n = 9; Trans-PAO; n = 9,Cis-PAO; n = 5 scale bar: 300 μm). **(E)** Macrophage content in aortic root lesions, as quantified after staining for MOMA-2 (green). (n = 5 per group, scale bar: 150 μm). In each case, a representative image is shown in left and center, and the quantification of the data appears on the right. Control versus trans-PAO or cis-PAO; Mann Whitney U test (in A B,C,D) Student's t test was used for statistical analysis.

We next investigated trans-PAO's role in atherosclerotic plaque development. Our previous studies showed cis-PAO can prevent hyperlipidemia-induced organelle stress, inflammasome activation, and inflammation and reduce atherosclerosis progression. However, in this study we observed that trans-PAO did not prevent PA-stimulated and inflammasome-produced IL-1β or other inflammatory cytokine production from macrophages *in vitro*. In fact, we observed some induction of cytokines with trans-PAO and PA co-stimulation of the macrophages (Figure 1D–E). Moreover, trans-PAO could not prevent SFA-induced ER or mitochondrial oxidative stress as cis-PAO does (Figure 1A–C). Therefore, our findings suggest that trans-PAO may be pro-inflammatory and pro-atherogenic, in contrast to the published epidemiological studies that imply a beneficial role for trans-PAO in cardiometabolic disease [4], [11], [12], [13]. While we observed effective trans-PAO enrichment in plasma and tissues of our mice (Figure 2, Figure 3), this was not accompanied with significant changes in body weight or blood glucose levels (S. Fig. 4A). Similarly, cis-PAO treatment in mice did not alter body weight or blood glucose levels (S. Fig. 4A). We then evaluated the impact of trans-PAO on atherosclerosis development in *en face* aorta preparations and aortic root sections. To our surprise, we found that trans-PAO treatment did not impact (neither preventing nor promoting) the development of atherosclerotic lesions in lesions in en face aorta preparations, whereas cis-PAO significantly reduces atherosclerosis (Figure 5A). Oil RedO staining analysis of the aortic root cryosections also did not show any significant changes in foamy macrophage accumulation in the aortic sinus area whereas cis-PAO significantly reduced foamy macrophages in lesions (Figure 5B). There were no significant differences in lesion area or the necrotic core area assessed from the hematoxylin and eosin (H&E)-stained lesions from trans-PAO-treated mice while cis-PAO significantly reduced necrotic core area (Figure 5C,D). Macrophage numbers in lesions were not reduced by trans-PAO whereas cis-PAO treatment resulted in significant drop in lesion macrophages (Figure 5E). Also, we noted no significant changes in the plasma total cholesterol or total triglycerides or the cholesterol content of lipoproteins between trans-PAO-treated and control mice, but we noted a small but significant increase in the TG amount found in plasma high density lipoprotein (S. Fig. 4B–F).

**Figure 6:**
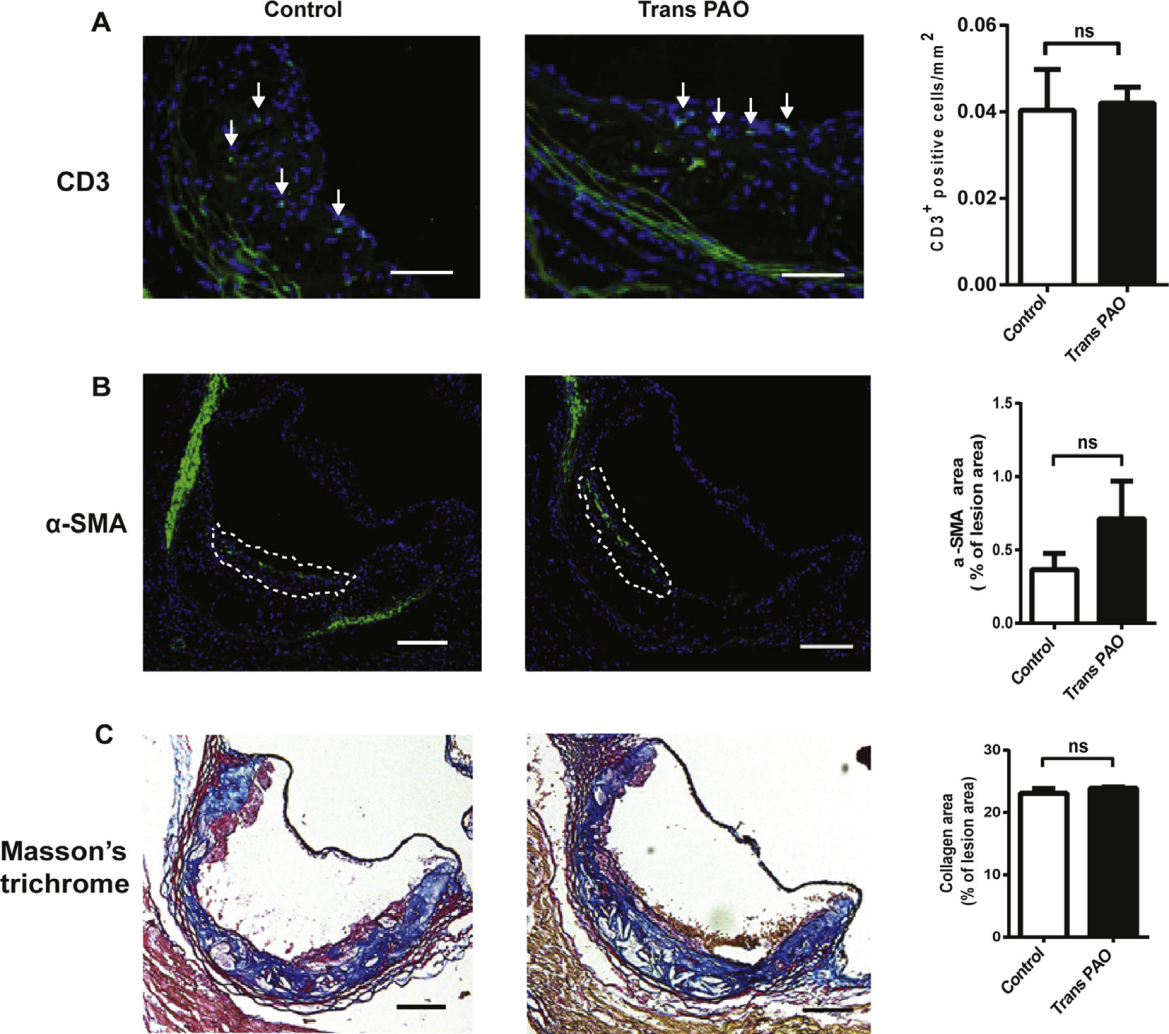
**Trans-PAO does not alter plaque composition of atherosclerotic lesions.** Immunohistochemical analysis of aortic root cryosections from Apoe^−/−^ mice from treated with trans-PAO or vehicle (1% BSA in PBS). The representative images and quantification are shown for: **(A)** CD3 (green), **(B)** α-SMA (green) **(C)** Masson's Trichrome staining (blue: collagen; red: cytoplasm and muscle fibers) (n = 5 per group). In each case, a representative image is shown in left and center, and the quantification of the data appears on the right. Data represents mean ± SEM; NS = not significant. Control versus trans-PAO; Mann Whitney U test was used for statistical analysis. Scale bars: 150 μm.

Vascular smooth muscle cells (SMC), endothelial cells, and immune cells, including lymphocytes, neutrophils, dendritic cells, and macrophages play important roles in the development of atherosclerotic plaques in the arterial wall [64]. Next, we performed immunohistochemical analysis of the aortic root sections from these mice in order to determine the impact of trans-PAO on plaque cellular composition. We observed that total T lymphocyte content did not differ between trans-PAO and control groups (Figure 6A). In addition, SMC content (assessed by α-smooth muscle actin staining) and collagen content (determined by Masson Trichrome staining) did not reflect significant differences between the lesions obtained from trans-PAO-treated mice and controls (Figure 6B and Figure 6C). Collectively, these findings demonstrate that trans-PAO treatment does not impact atherosclerosis development in mice.

## 4 Discussion

Owing to their effects on the plasma lipids, lipoprotein metabolism and inflammation, the quantity and type of dietary fatty acids have a considerable impact on the development of CVD [65]. Whereas MUFA with cis double bond configuration has been consistently associated with beneficial metabolic effects (such as anti-inflammatory, anti-diabetic and cardio protective actions) [63], MUFA with trans double bond configuration are often generated as by-products of industrially processed foods and have been associated with harmful effects on metabolism (such as inflammation and increased diabetes and CVD risk) [66], [67]. An exception to this appears to be ruminant-derived TFA such as vaccenic acid and trans-PAO, which have been associated with beneficial cardiometabolic outcomes [67]. Circulating trans-PAO represents less than one percent of total fatty acids (and significantly lower than cis-PAO levels) in humans. However, studies have shown a significant increase in this minor plasma trans-fatty acid mirrors full fatty dairy consumption and associates with lower TG, atherogenic dyslipidemia, and diabetes incidence [4], [12]. These beneficial attributes of trans-PAO are reminiscent of its isomer's (cis-PAO's) role in preventing insulin resistance, hepatic lipogenesis, and atherosclerosis [13], [23]. Despite having been implied by epidemiological studies as a beneficial TFA, a causal relationship between trans-PAO and metabolic health has not been experimentally demonstrated. In this study, we demonstrated trans-PAO, despite two to three folds enrichment in various tissues, has no direct impact on hyperlipidemia-induced inflammation and atherosclerosis progression in the Apoe^−/−^ mouse model of hypercholesterolemia-driven atherosclerosis.

SFA or cholesterol loading of macrophages is known to induce robust ER stress and mitochondrial oxidative stress, upstream of NLRP3 inflammasome activation and IL-1β and IL-18 secretion, resulting in an inflammatory response that drives atherosclerosis progression [23], [36], [59]. Genetic or pharmacological suppression of ER stress, mitochondrial oxidative stress and inflammasome activity reduces atherosclerosis [28], [36], [49], [68], [69], [70]. The results of the recent CANTOS trial showed neutralizing IL-1β with a human monoclonal antibody leads to dose-dependently reduction in C-reactive protein (CRP) and IL-6 levels (43% reduction from the baseline) in patients with previous myocardial infarction, providing support for the inflammatory basis of athero-thrombosis in humans [71]. Previous studies in humans also showed chronic cis-PAO administration reduced CRP and inflammation in humans. Further characterization of cis-PAO in mice demonstrated that this bioactive lipid prevents hyperlipidemia-induced ER stress, inflammasome activation, and inflammation, thereby reducing the progression of atherosclerosis. While trans-PAO has been associated with healthy cardiometabolic indicators in humans, we were surprised to discover that trans-PAO, unlike cis-PAO, could not antagonize SFA-induced organelle stress (as evidenced by unaltered UPR signaling and mtROS production) and inflammasome-induced IL-1β secretion in macrophages. Intriguingly, in cultured macrophages trans-PAO appeared to induce the production of TNFα and MCP1. Our findings are therefore in contrast to the epidemiological studies that have associated trans-PAO with reduced inflammation [12], [72]. Moreover, both of these cytokines were shown to be significantly inhibited by cis-PAO treatment in lipid-stressed mouse and human macrophages [23]. Therefore, in order to evaluate trans-PAO's impact on hyperlipidemia-induced organelle stress and inflammation in an *in vivo* setting, we administered trans-PAO orally to Apoe^−/−^ mouse model of atherosclerosis. Despite being partially pro-inflammatory in cultured macrophages, chronic trans-PAO treatment *in vivo* did not enhance nor reduce hyperlipidemia-induced atherosclerosis development in mice. Moreover, orally active trans-PAO did not reduce or induce hyperlipidemia-induced IL-1β levels in the plaques or in the serum of Apoe^−/−^ mice, which was consistent with our observations on trans-PAO's impact on IL-1β secretion in macrophages. In addition, trans-PAO did not alter macrophage accumulation or formation of foamy macrophages or necrotic core in lesions obtained from the aortic roots of Apoe^−/−^ mice. These *in vivo* findings therefore suggest ruminant-derived trans-PAO may not be the beneficial factor found in full fat dairy that appears to reduce the risk for CVD in humans [66]. Similar to our findings, others have shown that another ruminant TFA, trans-vaccenic acid (trans-18:1n–7), does not alter atherosclerosis development in rabbits and monkeys [73], [74]. In summary, results of this study support the findings from epidemiological studies that show trans-PAO does not increase the risk of CVD in humans, but it also shows that it doesn't possess the anti-inflammatory and atheroprotective properties attributed to its cis isomer. Whether trans-PAO has an impact on increasing insulin sensitivity requires further investigation in a diet-induced obesity mouse model as the Apoe^−/-^ mice do not represent a good model to study insulin resistance.

The cis isomer of PAO shows a protective effect against atherosclerosis through a major remodeling of intracellular organelle membranes. Both unsaturated and saturated fatty acids are potent regulators of membrane fluidity in part by their incorporation into phospholipids alters cholesterol affinity and incorporation into membranes [75]. This phenomenon can impact the activities of membrane proteins such as G-protein coupled receptors on the plasma membrane as much as the stress sensor/effector protein on the ER membrane (such as IRE1) [23], [75].

We previously showed that cis-PAO actively incorporates into major lipid classes in mice as well as isolated macrophages [23]. In this study, we observed that orally administered trans-PAO also incorporates into major lipids in plasma and membranes in tissues. Trans-PAO concentration is usually less than 1% of total lipids in humans [12], [13]. Our current lipidomics findings represent the first in depth analysis of trans-PAO in mice, revealing ∼1% of trans-PAO is found among lipid species in plasma and tissues of Apoe^−/−^ mice (fed with WD), whereas cis-PAO concentration is ∼10–15 folds more than its trans isomer. These results confirm that trans-PAO levels are significantly lower than cis-PAO isomer in mice. Although trans fatty acids are unsaturated, their pi bonds are not kinked (degree of acyl chain bending) compared to cis pi bonds. Thus, when incorporated into membranes, it is expected that they pack like saturated fatty acids and a smaller pool of trans fatty acids will be incorporated into the membranes [76]. Our data show that oral trans-PAO supplementation in hyperlipidemic mice leads to 2–3 folds trans-PAO enrichment in various tissues and plasma. Oral supplementation with cis-PAO in the hyperlipidemic mice leads to about 1.5 fold enrichment in various tissues. Albeit total trans-PAO levels remain much lower than normal cis-PAO levels, the fold enrichment of the trans isomer (2–3 fold) is bigger than the cis-isoform (about 1.5 fold). While cis-PAO enrichment is merely ∼1.5 fold in the examined tissues, its impact on inflammation and atheroprotection is profound [23]. The differential action of the two PAO isomers may involve their differential impact on the membrane desaturation index. A noticeable consequence of cis-PAO supplementation is the increase in systemic desaturation ratio in tissues, but trans-PAO treatment doesn't impact the systemic desaturation ratio in plasma or tissues in mice. Differential impact of cis-PAO and trans-PAO on the desaturation index can have important consequences for membranes. It is known that as a result of increased desaturation of membranes cis-PAO prevents SFA-induced IRE1 oligomerization on ER membranes [23]. Consistent with a lack of impact on the desaturation index, trans-PAO does not alter SFA-induced IRE oligomerization on ER membranes [23]. Because this trans fatty acid does not create the degree of acyl chain bending that cis does, the effect of the double bond on membrane physical properties could be greatly diminished [76]. Although our data show that trans-PAO can incorporate into the lipid pools leading to 2–3 fold enrichment of this trans-fatty acid, trans-PAO's effects on membrane desaturation, organelle stress and inflammation are muted due to its structure.

Plasma trans-PAO levels are regarded as a circulating fatty acid biomarker of full fat dairy consumption, which is associated with higher HDL-cholesterol and lower triglycerides concentrations [13], [77], [78]. In the present work, we found that chronic trans-PAO treatment did not influence LDL or HDL-cholesterol and triglycerides in plasma. Interestingly, trans-PAO treatment significantly increased HDL-triglyceride in plasma of Apoe^−/-^ mice, but this doesn't appear to promote atherosclerosis progression in mice. Our findings show that double bond configuration is critical for PAO's incorporation into lipids and beneficial metabolic properties such as reduction of organelle stress and anti-inflammatory actions that help prevent atherosclerosis. Of the two isoforms that we tested in our studies, only orally active cis-PAO reduced organelle stress, inflammation, and atherosclerosis [23]. Trans-PAO neither prevented nor promoted atherogenesis in mice. Therefore, our findings support previous epidemiological studies that found elevated trans-PAO levels, despite being a TFA, does not increase risk for CVD [79].

## 5 Conclusion

Our findings demonstrate the importance of comprehensive and direct investigation of lipids, which have been associated with metabolic benefits in epidemiological studies, in appropriate disease models. The results of our two studies on cis-PAO and trans-PAO will provide useful guidance for future dietary intervention strategies in humans that are based on PAO supplementation. Our findings show trans-PAO is not atheroprotective like cis-PAO but also confirm epidemiological studies that showed trans-PAO does not promote CVD.

## References

[1] Wang D.D., Hu F.B., Stover P.J., Balling R. (2017). Dietary fat and risk of cardiovascular disease: recent controversies and advances.

[2] Brouwer I.A., Wanders A.J., Katan M.B. (2013). Trans fatty acids and cardiovascular health: research completed?. European Journal of Clinical Nutrition.

[3] Mozaffarian D., Katan M.B., Ascherio A., Stampfer M.J., Willett W.C. (2006). Medical progress - trans fatty acids and cardiovascular disease. New England Journal of Medicine.

[4] Kleber M.E., Delgado G.E., Lorkowski S., Marz W., von Schacky C. (2016). Trans-fatty acids andmortality in patients referred for coronary angiography: the Ludwigshafen Risk and Cardiovascular Health Study. European Heart Journal.

[5] Willett W.C., Stampfer M.J., Manson J.E., Colditz G.A., Speizer F.E., Rosner B.A. (1993). Intake of trans-fatty-acids and risk of coronary heart-disease among women. Lancet.

[6] Sun Q., Ma J., Campos H., Hankinson S.E., Manson J.E., Stampfer M.J. (2007). A prospective study of Trans fatty acids in erythrocytes and risk of coronary heart disease. Circulation.

[7] Chien K.L., Lin H.J., Hsu H.C., Chen P.C., Su T.C., Chen M.F. (2013). Comparison of predictive performance of various fatty acids for the risk of cardiovascular disease events and all-cause deaths in a community-based cohort. Atherosclerosis.

[8] Laake I., Pedersen J.I., Selmer R., Kirkhus B., Lindman A.S., Tverdal A. (2012). A prospective study of intake of trans-fatty acids from ruminant fat, partially hydrogenated vegetable oils, and marine oils and mortality from CVD. British Journal of Nutrition.

[9] Dyer O. (2012). Bans and labelling helped to reduce Americans' trans fat levels by 58%. British Medical Journal.

[10] Kuhnt K., Degen C., Jahreis G. (2016). Evaluation of the impact of ruminant trans fatty acids on human health: important aspects to consider. Critical Reviews in Food Science and Nutrition.

[11] Kratz M., Marcovina S., Nelson J.E., Yeh M.M., Kowdley K.V., Callahan H.S. (2014). Dairy fat intake is associated with glucose tolerance, hepatic and systemic insulin sensitivity, and liver fat but not beta-cell function in humans. American Journal of Clinical Nutrition.

[12] Mozaffarian D., Cao H.M., King I.B., Lemaitre R.N., Song X.L., Siscovick D.S. (2010). Trans-Palmitoleic acid, metabolic risk factors, and new-onset diabetes in U.S. Adults a cohort study. Annals of Internal Medicine.

[13] Mozaffarian D., Otto M.C.D., Lemaitre R.N., Fretts A.M., Hotamisligil G., Tsai M.Y. (2013). trans-Palmitoleic acid, other dairy fat biomarkers, and incident diabetes: the Multi-Ethnic Study of Atherosclerosis (MESA). American Journal of Clinical Nutrition.

[14] Santaren I.D., Watkins S.M., Liese A.D., Wagenknecht L.E., Rewers M.J., Haffner S.M. (2014). Serum pentadecanoic acid (15:0), a short-term marker of dairy food intake, is inversely associated with incident type 2 diabetes and its underlying disorders. American Journal of Clinical Nutrition.

[15] Kroger J., Zietemann V., Enzenbach C., Weikert C., Jansen E., Doring F. (2011). Erythrocyte membrane phospholipid fatty acids, desaturase activity, and dietary fatty acids in relation to risk of type 2 diabetes in the European Prospective Investigation into Cancer and Nutrition (EPIC)-Potsdam Study. American Journal of Clinical Nutrition.

[16] Patel P.S., Sharp S.J., Jansen E., Luben R.N., Khaw K.T., Wareham N.J. (2010). Fatty acids measured in plasma and erythrocyte-membrane phospholipids and derived by food-frequency questionnaire and the risk of new-onset type 2 diabetes a pilot study in the European Prospective Investigation into Cancer and Nutrition (EPIC)-Norfolk co. American Journal of Clinical Nutrition.

[17] Castro-Webb N., Ruiz-Narvaez E.A., Campos H. (2012). Cross-sectional study of conjugated linoleic acid in adipose tissue and risk of diabetes. American Journal of Clinical Nutrition.

[18] Cao H., Gerhold K., Mayers J.R., Wiest M.M., Watkins S.M., Hotamisligil G.S. (2008). Identification of a lipokine, a lipid hormone linking adipose tissue to systemic metabolism. Cell.

[19] Dimopoulos N., Watson M., Sakamoto K., Hundal H.S. (2006). Differential effects of palmitate and palmitoleate on insulin action and glucose utilization in rat L6 skeletal muscle cells. Biochemical Journal.

[20] Sauma L., Stenkula K.G., Kjolhede P., Stralfors P., Soderstrom M., Nystrom F.H. (2006). PPAR-gamma response element activity in intact primary human adipocytes: effects of fatty acids. Nutrition.

[21] Maedler K., Oberholzer J., Bucher P., Spinas G.A., Donath M.Y. (2003). Monounsaturated fatty acids prevent the deleterious effects of palmitate and high glucose on human pancreatic beta-cell turnover and function. Diabetes.

[22] Erbay E., Babaev V.R., Mayers J.R., Makowski L., Charles K.N., Snitow M.E. (2009). Reducing endoplasmic reticulum stress through a macrophage lipid chaperone alleviates atherosclerosis. Nature Medicine.

[23] Cimen I., Kocaturk B., Koyuncu S., Tufanl O., Onat U.I., Y ld r m A.D. (2016). Prevention of atherosclerosis by bioactive palmitoleate through suppression of organelle stress and inflammasome activation. Science Translational Medicine.

[24] Bernstein A.M., Roizen M.F., Martinez L. (2014). Purified palmitoleic acid for the reduction of high-sensitivity C-reactive protein and serum Lipids: a double-blinded, randomized, placebo controlled study. Journal of Clinical Lipidology.

[25] Yilmaz M., Claiborn K.C., Hotamisligil G.S. (2016). De novo lipogenesis products and endogenous lipokines. Diabetes.

[26] Tabas I. (2017). 2016 russell ross memorial lecture in vascular biology molecular-cellular mechanisms in the progression of atherosclerosis. Arteriosclerosis, Thrombosis, and Vascular Biology.

[27] Tabas I., Bornfeldt K.E. (2016). Macrophage phenotype and function in different stages of atherosclerosis. Circulation Research.

[28] Duewell P., Kono H., Rayner K.J., Sirois C.M., Vladimer G., Bauernfeind F.G. (2010). NLRP3 inflammasomes are required for atherogenesis and activated by cholesterol crystals. Nature.

[29] Libby P., Lichtman A.H., Hansson G.K. (2013). Immune effector mechanisms implicated in atherosclerosis: from mice to humans. Immunity.

[30] Wen H.T., Gris D., Lei Y., Jha S., Zhang L., Huang M.T.H. (2011). Fatty acid-induced NLRP3-ASC inflammasome activation interferes with insulin signaling. Nature Immunology.

[31] He Y., Hara H., Nunez G. (2016). Mechanism and regulation of NLRP3 inflammasome activation. Trends in Biochemical Sciences.

[32] Hotamisligil G.S., Erbay E. (2008). Nutrient sensing and inflammation in metabolic diseases. Nature Reviews Immunology.

[33] Feng B., Yao P.M., Li Y., Devlin C.M., Zhang D., Harding H.P. (2003). The endoplasmic reticulum is the site of cholesterol-induced cytotoxicity in macrophages. Nature Cell Biology.

[34] Gora S., Maouche S., Atout R., Wanherdrick K., Lambeau G., Cambien F. (2010). Phospholipolyzed LDL induces an inflammatory response in endothelial cells through endoplasmic reticulum stress signaling. The FASEB Journal.

[35] Xu C.Y., Bailly-Maitre B., Reed J.C. (2005). Endoplasmic reticulum stress: cell life and death decisions. Journal of Clinical Investigation.

[36] Tufanli O., Telkoparan Akillilar P., Acosta-Alvear D., Kocaturk B., Onat U.I., Hamid S.M. (2017). Targeting IRE1 with small molecules counteracts progression of atherosclerosis. Proceedings of the National Academy of Sciences.

[37] Verfaillie T., Rubio N., Garg A.D., Bultynck G., Rizzuto R., Decuypere J.P. (2012). PERK is required at the ER-mitochondrial contact sites to convey apoptosis after ROS-based ER stress. Cell Death & Differentiation.

[38] Gregor M.F., Hotamisligil G.S. (2011). Inflammatory mechanisms in obesity. Annual Review of Immunology.

[39] Horng T. (2014). Calcium signaling and mitochondrial destabilization in the triggering of the NLRP3 inflammasome. Trends in Immunology.

[40] Walter P., Ron D. (2011). The unfolded protein response: from stress pathway to homeostatic regulation. Science.

[41] Park S.W., Ozcan U. (2013). Potential for therapeutic manipulation of the UPR in disease. Seminars in Immunopathology.

[42] Ozcan U., Yilmaz E., Ozcan L., Furuhashi M., Vaillancourt E., Smith R.O. (2006). Chemical chaperones reduce ER stress and restore glucose homeostasis in a mouse model of type 2 diabetes. Science.

[43] Ozcan U., Ozcan L., Yilmaz E., Duvel K., Sahin M., Manning B.D. (2008). Loss of the tuberous sclerosis complex tumor suppressors triggers the unfolded protein response to regulate insulin signaling and apoptosis. Molecular Cell.

[44] Ron D., Walter P. (2007). Signal integration in the endoplasmic reticulum unfolded protein response. Nature Reviews Molecular Cell Biology.

[45] Harding H.P., Zhang Y.H., Ron D. (1999). Protein translation and folding are coupled by an endoplasmic-reticulum-resident kinase (vol 397, pg 271, 1999). Nature.

[46] Schroder M., Kaufman R.J. (2005). The mammalian unfolded protein response. Annual Review of Biochemistry.

[47] Halbleib K., Pesek K., Covino R., Hofbauer H.F., Wunnicke D., Hanelt I. (2017). Activation of the unfolded protein response by lipid bilayer stress. Molecular Cell.

[48] Volmer R., van der Ploeg K., Ron D. (2013). Membrane lipid saturation activates endoplasmic reticulum unfolded protein response transducers through their transmembrane domains. Proceedings of the National Academy of Sciences of the United States of America.

[49] Onat U.I., Yildirim A.D., Tufanli Ö., Çimen I., Kocatürk B., Veli Z. (2019). Intercepting the lipid-induced integrated stress response reduces atherosclerosis. Journal of the American College of Cardiology.

[50] Robblee M.M., Kim C.C., Abate J.P., Valdearcos M., Sandlund K.L.M., Shenoy M.K. (2016). Saturated fatty acids engage an IRE1 alpha-dependent pathway to activate the NLRP3 inflammasome in myeloid cells. Cell Reports.

[51] Li H., Korennykh A.V., Behrman S.L., Walter P. (2010). Mammalian endoplasmic reticulum stress sensor IRE1 signals by dynamic clustering. Proceedings of the National Academy of Sciences of the United States of America.

[52] Han S., Liang C.P., DeVries-Seimon T., Ranalletta M., Welch C.L., Collins-Fletcher K. (2006). Macrophage insulin receptor deficiency increases ER stress-induced apoptosis and necrotic core formation in advanced atherosclerotic lesions. Cell Metabolism.

[53] Seimon T.A., Wang Y., Han S., Senokuchi T., Schrijvers D.M., Kuriakose G. (2009). Macrophage deficiency of p38alpha MAPK promotes apoptosis and plaque necrosis in advanced atherosclerotic lesions in mice. Journal of Clinical Investigation.

[54] Döring Y., Noels H., van der Vorst E.P.C., Neideck C., Egea V., Drechsler M. (2017). Vascular CXCR4 limits atherosclerosis by maintaining arterial integrity. Circulation.

[55] Rinne P., Rami M., Nuutinen S., Santovito D., van der Vorst E.P.C., Guillamat-Prats R. (2017). Melanocortin 1 receptor signaling regulates cholesterol transport in macrophages. Circulation.

[56] Doran A.C., Ozcan L., Cai B.S., Zheng Z., Fredman G., Rymond C.C. (2017). CAMKII gamma suppresses an efferocytosis pathway in macrophages and promotes atherosclerotic plaque necrosis. Journal of Clinical Investigation.

[57] Diakogiannaki E., Morgan N.G. (2008). Differential regulation of the ER stress response by long-chain fatty acids in the pancreatic beta-cell. Biochemical Society Transactions.

[58] Guo H.T., Callaway J.B., Ting J.P.Y. (2015). Inflammasomes: mechanism of action, role in disease, and therapeutics. Nature Medicine.

[59] Chan K.L., Pillon N.J., Sivaloganathan D.M., Costford S.R., Liu Z., Theret M. (2015). Palmitoleate reverses high fat-induced proinflammatory macrophage polarization via AMP-activated protein kinase (AMPK). Journal of Biological Chemistry.

[60] Niu S.L., Mitchell D.C., Litman B.J. (2005). Trans fatty acid derived phospholipids show increased membrane cholesterol and reduced receptor activation as compared to their cis analogs. Biochemistry.

[61] Libby P. (2012). Inflammation in atherosclerosis. Arteriosclerosis, Thrombosis, and Vascular Biology.

[62] Babaev V.R., Yeung M., Erbay E., Ding L., Zhang Y.M., May J.M. (2016). Jnk1 deficiency in hematopoietic cells suppresses macrophage apoptosis and increases atherosclerosis in low-density lipoprotein receptor null mice. Arteriosclerosis, Thrombosis, and Vascular Biology.

[63] Hirata Y., Takahashi M., Kudoh Y., Kano K., Kawana H., Makide K. (2017). trans-Fatty acids promote proinflammatory signaling and cell death by stimulating the apoptosis signal-regulating kinase 1 (ASK1)-p38 pathway. Journal of Biological Chemistry.

[64] Tabas I. (2010). The role of endoplasmic reticulum stress in the progression of atherosclerosis. Circulation Research.

[65] Machado R.M., Nakandakare E.R., Quintao E.C., Cazita P.M., Koike M.K., Nunes V.S. (2013). Omega-6 polyunsaturated fatty acids prevent atherosclerosis development in LDLr-KO mice, in spite of displaying a pro-inflammatory profile similar to trans fatty acids (vol 224, pg 66, 2012). Atherosclerosis.

[66] Estadella D., do Nascimento C., Oyama L.M., Ribeiro E.B., Damaso A.R., de Piano A. (2013). Lipotoxicity: effects of dietary saturated and transfatty acids. Mediators of Inflammation.

[67] Nettleton J.A., Lovegrove J.A., Mensink R.P., Schwab U. (2016). Dietary fatty acids: is it time to change the recommendations?. Annals of Nutrition and Metabolism.

[68] Abderrazak A., Couchie D., Mahmood D.F.D., Elhage R., Vindis C., Laffargue M. (2015). Anti-inflammatory and antiatherogenic effects of the NLRP3 inflammasome inhibitor arglabin in ApoE(2).Ki mice fed a high-fat diet. Circulation.

[69] Sheedy F.J., Grebe A., Rayner K.J., Kalantari P., Ramkhelawon B., Carpenter S.B. (2013). CD36 coordinates NLRP3 inflammasome activation by facilitating intracellular nucleation of soluble ligands into particulate ligands in sterile inflammation. Nature Immunology.

[70] Wang Y., Wang G.Z., Rabinovitch P.S., Tabas I. (2014). Macrophage mitochondrial oxidative stress promotes atherosclerosis and nuclear factor-kappa B-mediated inflammation in macrophages. Circulation Research.

[71] Ridker P.M., Everett B.M., Thuren T., MacFadyen J.G., Chang W.H., Ballantyne C. (2017). Antiinflammatory therapy with canakinumab for atherosclerotic disease. New England Journal of Medicine.

[72] Yakoob M.Y., Shi P.L., Willett W.C., Rexrode K.M., Campos H., Orav E.J. (2016). Circulating biomarkers of dairy fat and risk of incident diabetes mellitus among men and women in the United States in two large prospective cohorts. Circulation.

[73] Ruttenberg H., Davidson L.M., Little N.A., Klurfeld D.M., Kritchevsky D. (1983). Influence of trans unsaturated fats on experimental atherosclerosis in rabbits. Journal of Nutrition.

[74] Kritchevsky D., Davidson L.M., Weight M., Kriek N.P.J., Duplessis J.P. (1984). Effect of trans-unsaturated fats on experimental atherosclerosis in vervet monkeys. Atherosclerosis.

[75] Ganguly R., Pierce G.N. (2012). Trans fat involvement in cardiovascular disease. Molecular Nutrition & Food Research.

[76] Roach C., Feller S.E., Ward J.A., Shaikh S.R., Zerouga M., Stillwell W. (2004). Comparison of Cis and Trans fatty acid containing phosphatidylcholines on membrane properties. Biochemistry.

[77] Sun Q., Ma J., Campos H., Hu F.B. (2007). Plasma and erythrocyte biomarkers of dairy fat intake and risk of ischemic heart diseased. American Journal of Clinical Nutrition.

[78] Jaudszus A., Kramer R., Pfeuffer M., Roth A., Jahreis G., Kuhnt K. (2014). Trans Palmitoleic acid arises endogenously from dietary vaccenic acid. American Journal of Clinical Nutrition.

[79] Liang J.J., Zhou Q., Amakye W.K., Su Y.X., Zhang Z.Q. (2018). Biomarkers of dairy fat intake and risk of cardiovascular disease: a systematic review and meta analysis of prospective studies. Critical Reviews in Food Science and Nutrition.

