# Supplementary material for: Double bond configuration of palmitoleate is critical for atheroprotection
Source: Mol Metab. 2019 Aug 7;28:58–72. doi: 10.1016/j.molmet.2019.08.004 (PMC6822256; doi:10.1016/j.molmet.2019.08.004)
Supplement: Multimedia component 1 [file mmc1.pdf]

SUPPLEMENTARY FILE:

Double Bond Configuration of Palmitoleate is Critical For Atheroprotection

Ismail Cimen[1], Zehra Yildirim[2,3,4,5], Asli Ekin Dogan[2,3,4,5], Asli Dilber Yildirim[2,3,4,5,], Özlem Tufanlı[6], Umut Inci Onat[2,3], UyenThao Nguyen[7] Steven M. Watkins[8], Christian Weber[1,9], and Ebru Erbay*[,2,3,4,5,10]

[1] Institute for Cardiovascular Prevention, LMU Munich, German Cardiovascular Research Centre (DZHK), partner site Munich Heart Alliance Munich, 80336, Germany

[2] Department of Molecular Biology and Genetics, Bilkent University, Ankara, 06800, Turkey

[3] National Nanotechnology Center, Bilkent University, Ankara, 06800, Turkey

[4] Department of Biomedical Sciences, Cedars-Sinai Medical Center, Los Angeles, CA, 90048, USA

[5] Smidt Heart Institute, Cedars-Sinai Medical Center, Los Angeles, CA, 90048, USA

[6] New York University, Lagone Medical Center, New York, NY 10016, USA

[7] Metabolon, Morrisville, North Carolina, 27560, USA

[8]Verso Biosciences, San Francisco, CA, 94124, USA

[9] Department of Biochemistry, Cardiovascular Research Institute Maastricht (CARIM), Maastricht, Maastricht University, The Netherlands

[10] David Geffen School of Medicine, University of California, Los Angeles, CA, 90095, USA

<u>* To whom correspondence should be addressed:</u>

Ebru Erbay, M.D., Ph.D.

Associate Professor

Cedars Sinai Medical Center

Department of Medicine, Heart Institute & Department of Biomedical Sciences

127 S. San Vincente Boulevard, Advanced Health Sciences Pavillion, A9104

Los Angeles, CA 90048

E-mail: ebru.erbay@cshs.org

Phone: (310) 4237483

Supplementary material

Supplementary Figure 1

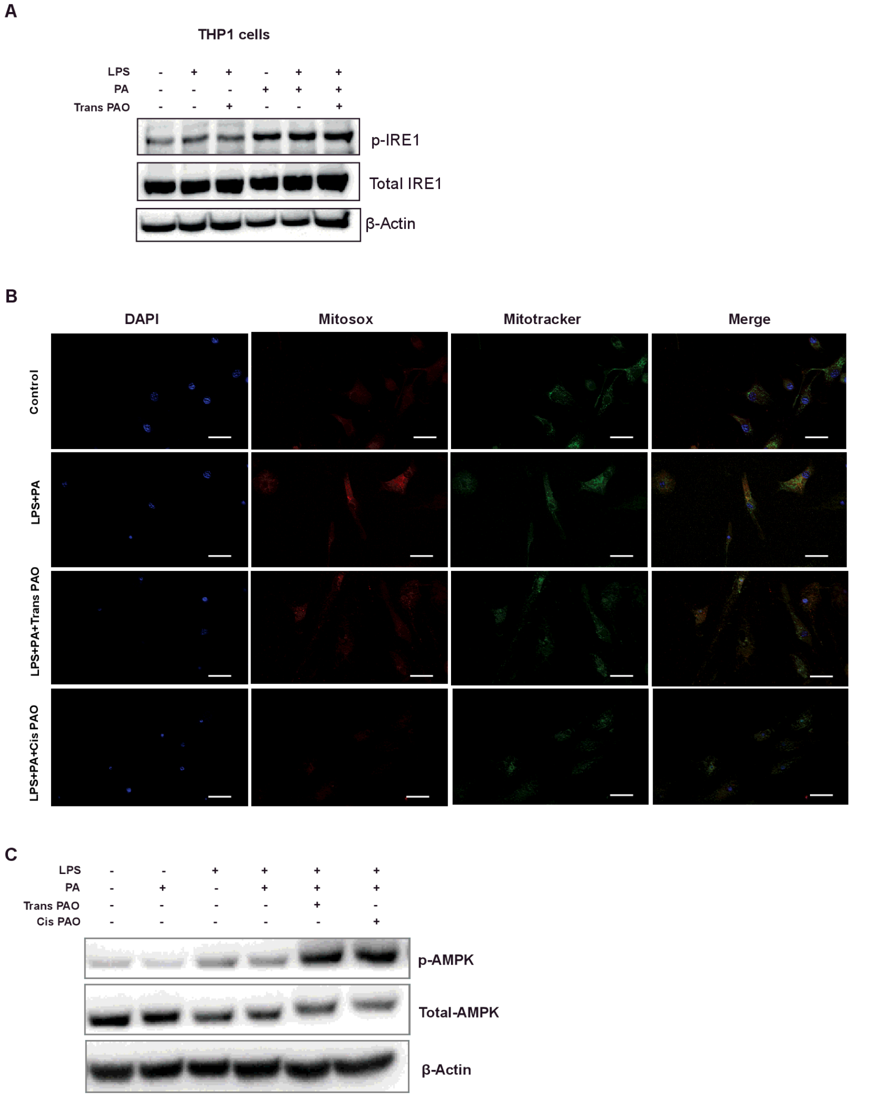

S.Figure 1. The impact of Trans-PAO treatment on lipid-induced ER and mitochondrial oxidative stress and AMPK activation. (A) LPS-primed, PA-stressed human THP1 macrohages were treated trans-PAO and protein lysates were analyzed by western blotting using specific antibodies against: **(A)** p-IRE1 and β-actin (n=3, a representative blot is shown). **(B)** Representative confocal images for lipid-induced mtROS production in LPS-primed and PA-stressed BMDMs, which were co-treated with trans- or cis-PAO (graphs for the respective mtROS quantifications were shown in Fig.1C). Mitosox Fluorescent Indicator: red; Mitotracker: green. **(C)** LPS-primed, PA-stressed BMDM were treated with trans- or cis-PAO and protein lysates were analyzed by western blotting using specific antibodies against: p-AMPK, total-AMPK and β-actin (n=3, a representative blot is shown).

Supplementary Figure 2

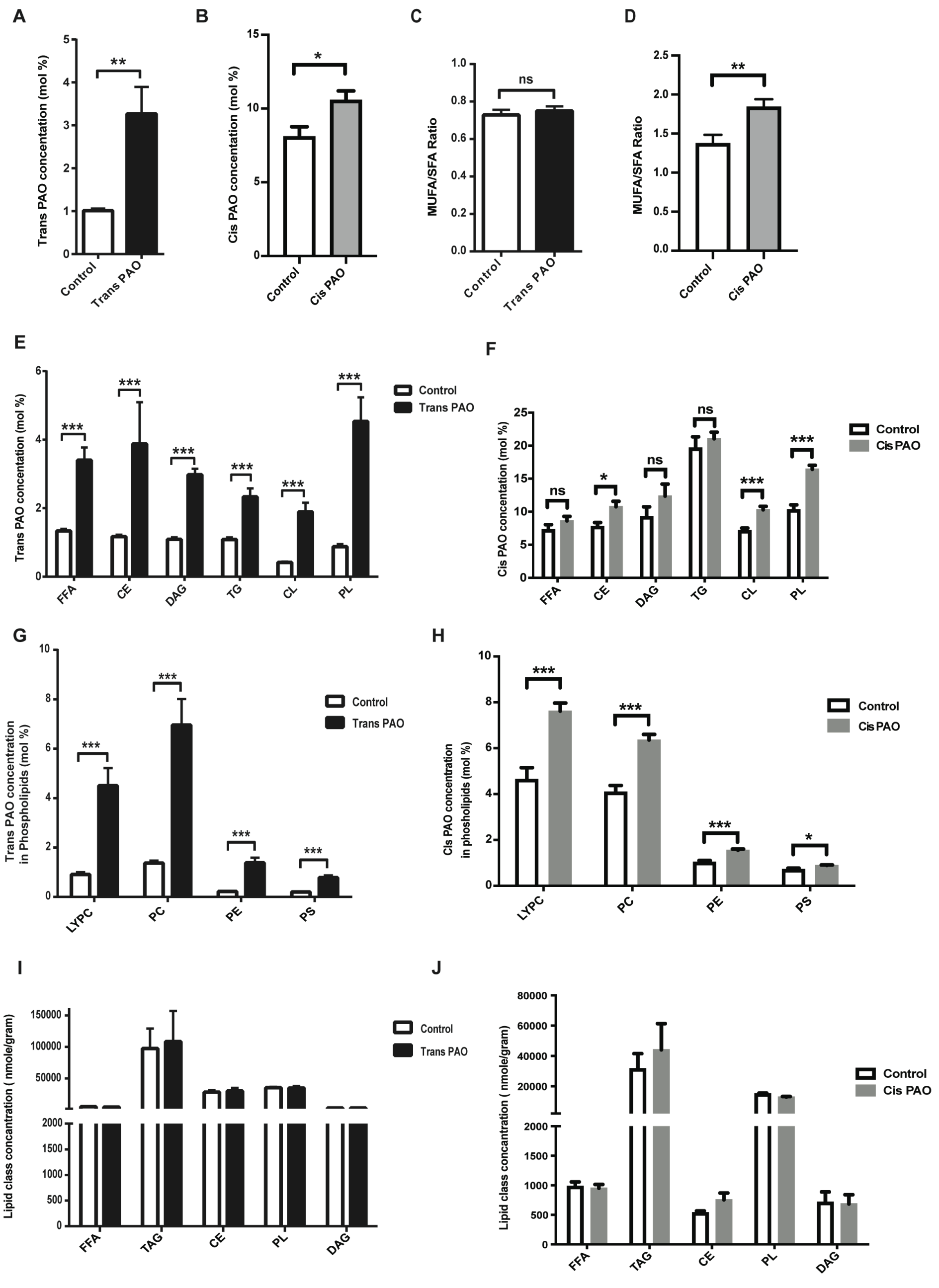

S. Figure 2. The effect of trans-PAO and cis-PAO oral supplementation on muscle lipid composition in the Apoe$^{-/-}$ mice. Quantiative lipidomic analysis was performed on skeletal muscle isolated from trans- or cis-PAO-treated and control Apoe$^{-/-}$ mice on WD: **(A,B)** The mean concentration of trans- or Cis-C16:1n-7 (mole %), **(C,D)** MUFA/SFA ratio after trans-PAO or cis-PAO treatment, **(E, F)** The mean concentration (mole %) of trans- or cis-C16:1n-7 in various lipid classes. (**G,H**) The mean concentration (mole %) of trans- or cis-C16:1n-7 in the various phospholipid classes. (**I,J**) The mean concentration (mole/gram) of various lipid classes in trans- or cis-PAO treated and control mice. Data represents mean ± SEM; *P< 0.05, **P<0.01, **P<0.001, ns = not significant, (n=5 for trans-PAO; n=6 for cis-PAO). Unpaired two-tailed Student's t test was used for statistical analysis.

Supplementary Figure 3

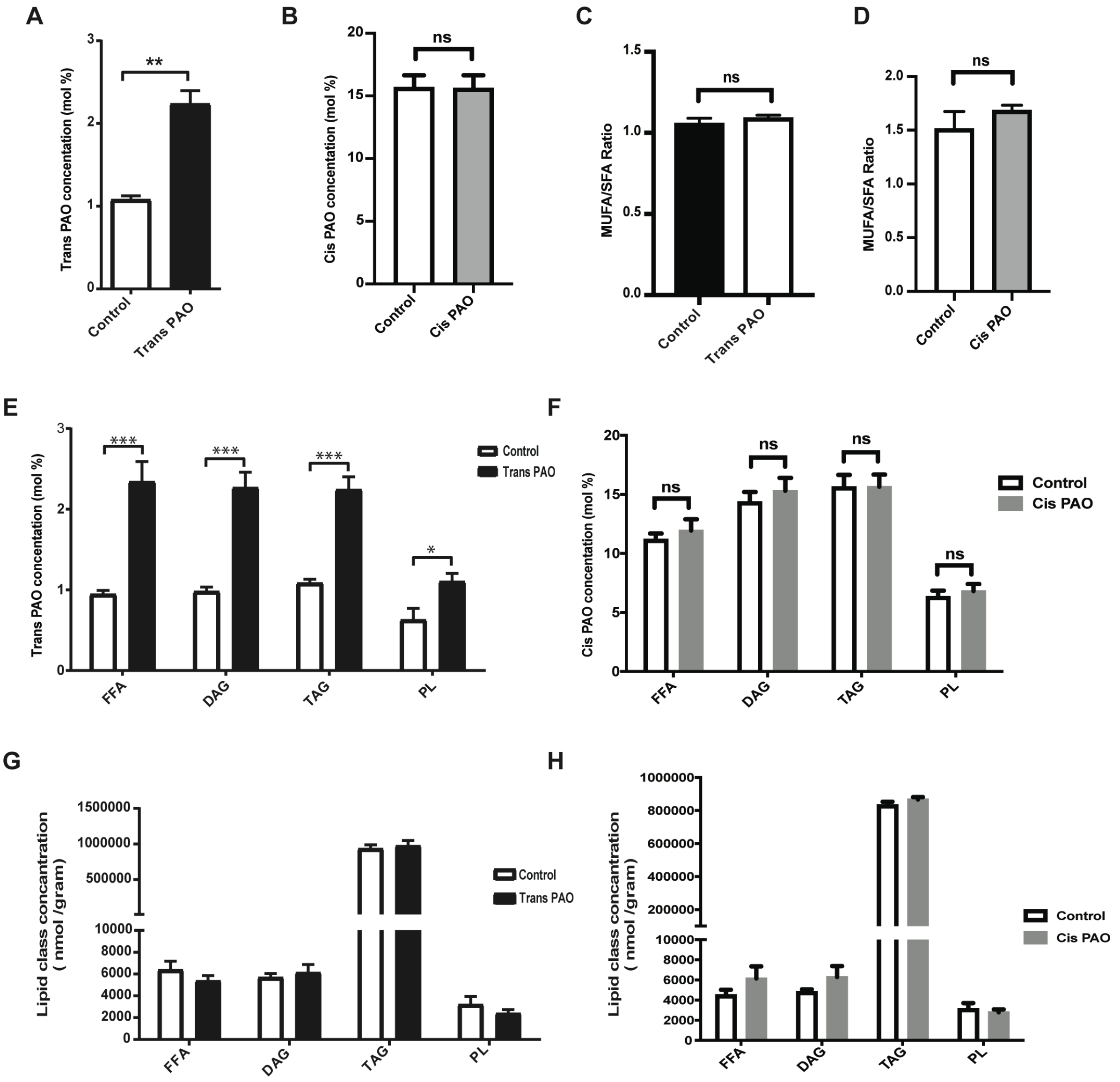

S.Figure 3. The effect of trans-PAO or cis-PAO oral supplementation on adipose tissue lipid composition in the Apoe$^{-/-}$ mice. Quantitative lipidomic analysis of the adipose tissue from trans- or cis-PAO treated and control Apoe$^{-/-}$ mice on WD: **(A, B)** The mean concentration of trans- or cis-C16:1n-7 (mole %). **(C,D)** MUFA/SFA ratio after trans-PAO or cis-PAO treatment. **(E, F)** The mean concentration (mole %) of trans- or cis-C16:1n-7 in various lipid classes. **(G,H)** The mean concentration (mole/gram) of various lipid classes in trans- or cis-PAO-treated and control mice. Data represents mean ± SEM; *P<0.05, **P<0.01, **P<0.001, ns = not significant, (n=5 for trans-PAO; n=6 for cis-PAO). Unpaired two-tailed Student's t test was used for statistical analysis.

Supplementary Figure 4

A. Physical and biochemical characteristic of Apoe-/- mice in atherosclerosis studies

Variables	Treatment	Control	Trans-PAO	Cis PAO
n		12	9	6
Bodyweight (g)	Before	30.25±0.74	32.67±0.47	29±1.06
	After	30.92±0.82	31.89±061	28.67±061
Glucose (mg/dl)	Before	92.42±4.72	82.33±4.98	103.33±4.29
	After	88.00±4.51	91.78±6.36	93.67±7.29

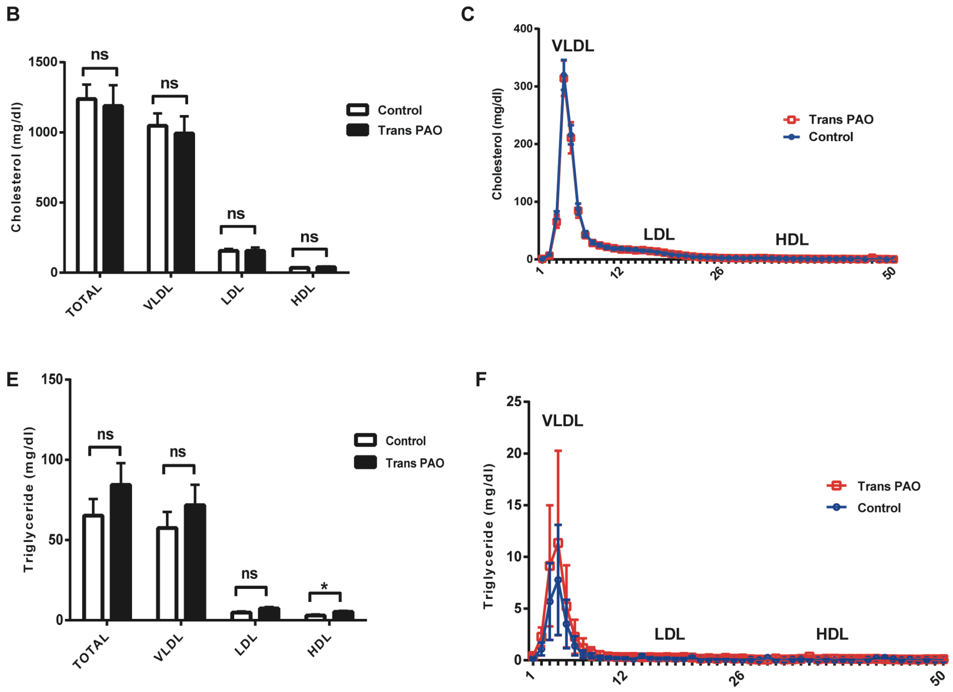

S.Figure 4. Physical and biochemical characteristics of trans-PAO-treated Apoe$^{-/-}$ mice. Measurements are from Apoe$^{-/-}$ mice on WD that received oral supplementation with PAO: **(A)** Total blood glucose and body weight changes, **(B)** Total plasma cholesterol and lipoprotein (VLDL, LDL, HDL) cholesterol levels. **(C)** Lipoprotein profiles from control (blue) and trans-PAO-treated (red) Apoe$^{-/-}$ mice show an average percent distribution of total cholesterol for each group (n=5 per group). **(D)**Total plasma triglyceride and lipoprotein (VLDL, LDL, HDL) triglyceride levels. **(E)** Lipoprotein profiles from control (blue) and trans-PAO-treated (red) Apoe$^{-/-}$ mice show an average percent distribution of total triglyceride for each group (n=5 per group). Data are expressed as the mean ± SEM. VLDL, very low density lipoprotein; LDL, low density lipoprotein; HDL, high density lipoprotein. Data are expressed as the mean ± SEM. ns = not significant *P < 0.05, (n=5 per group). Unpaired two-tailed Student’s t test was used for statistical analysis.
